# Metagenomic insights into the rumen microbial fibrolytic enzymes in Indian crossbred cattle fed finger millet straw

**DOI:** 10.1186/s13568-016-0310-0

**Published:** 2017-01-03

**Authors:** V. Lyju Jose, Thulasi Appoothy, Ravi P. More, A. Sha Arun

**Affiliations:** 1 Rumen Microbiology Laboratory, Animal Nutrition Division, National Institute of Animal Nutrition and Physiology, Bangalore, India; 2 Department of Biotechnology, Jain University, Bangalore, India; 3 Bioinformatics Laboratory, Division of Molecular Entomology, National Bureau of Agricultural Insect Resources, Bangalore, India; 4 Wildlife SOS, Bannerghatta Bear Rescue Centre, Bangalore, India

**Keywords:** Rumen, Fibrolytic enzymes, Plant polysaccharides, Metagenomics, Microbiome

## Abstract

The rumen is a unique natural habitat, exhibiting an unparalleled genetic resource of fibrolytic enzymes of microbial origin that degrade plant polysaccharides. The objectives of this study were to identify the principal plant cell wall-degrading enzymes and the taxonomic profile of rumen microbial communities that are associated with it. The cattle rumen microflora and the carbohydrate-active enzymes were functionally classified through a whole metagenomic sequencing approach. Analysis of the assembled sequences by the Carbohydrate-active enzyme a﻿nalysis Toolkit identified the candidate genes encoding fibrolytic enzymes belonging to different classes of glycoside hydrolases(11,010 contigs), glycosyltransferases (6366 contigs), carbohydrate esterases (4945 contigs), carbohydrate-binding modules (1975 contigs), polysaccharide lyases (480 contigs), and auxiliary activities (115 contigs). Phylogenetic analysis of CAZyme encoding contigs revealed that a significant proportion of CAZymes were contributed by bacteria belonging to genera *Prevotella*, *Bacteroides, Fibrobacter, Clostridium,* and *Ruminococcus*. The results indicated that the cattle rumen microbiome and the CAZymes are highly complex, structurally similar but compositionally distinct from other ruminants. The unique characteristics of rumen microbiota and the enzymes produced by resident microbes provide opportunities to improve the feed conversion efficiency in ruminants and serve as a reservoir of industrially important enzymes for cellulosic biofuel production.

**Electronic supplementary material:**

The online version of this article (doi:10.1186/s13568-016-0310-0) contains supplementary material, which is available to authorized users.

## Introduction

Ruminants represent a substantial proportion of domesticated animal species worldwide and are the main source of milk, meat, and other dairy products. Ruminants have the ability to digest large amounts of plant polysaccharides by virtue of the composite microflora present in the rumen. The rumen has evolved into an efficient and effective fermentation vat for fiber degradation and the rumen is inhabited by a consortium of microorganisms consisting of bacteria, archaea, fungi, protozoa, and viruses (Miron et al. 2001; Pope et al. 2012), which interact and contribute significantly towards the health of ruminants. Among the various domains of microorganisms resident in the rumen, bacteria are predominant representing about 95% of the total microbes (Mackie et al. 2000; Lin et al. 1997). The rumen fermentation process mediated by microbial communities affects the quality and composition of milk and meat and the productive performance of the host (Welkie et al. 2010; Stevenson and Weimer 2007; Sundset et al. 2009).

In tropical countries like India, ruminants are primarily fed on lignocellulose based agricultural crop residues. The extensive rumen microbiota are endowed with the potential to hydrolyze the plant polymers into simpler forms that provides nutrients to the host, predominantly in the form of volatile fatty acids and microbial proteins. The rumen essentially functioning as an anaerobic fermenter, has the ability to absorb the digested plant polysaccharides by the resident microflora (Jami and Mizrahi 2012). Studies on the symbiotic rapport between the rumen microbial communities and the mammalian host have posed a challenging area of research for the scientific community in the past due to the lack of adequate techniques to investigate and analyse such complex ecosystem.

Rumen microbes produce an array of fibrolytic enzymes called Carbohydrate-Active Enzymes (CAZymes), including exoglucanases, endoglucanases, glucosidases, and hemicellulases to deal with the complex plant polysaccharides. High Throughput Sequencing (HTS) technologies are extensively used to address the intricate process of lignocellulose degradation in ruminants. An improved understanding of the rumen microbial ecosystem could address the challenges in ruminant nutrition and environmental concerns in the livestock sector. Numerous metagenomic studies have reported on the diversity of fibrolytic enzymes from the rumen of yak (Dai et al. 2012), reindeer (Pope et al. 2012), Jersey cow (Wang et al. 2013), Angus cattle (Brulc et al. 2009), and buffalo (Singh et al. 2014). However, there are no comprehensive scientific reports available on metagenomic studies on the rumen CAZymes profile of Holstein–Friesian crossbred cattle, fed only finger millet straw. Due to the paucity of information on the CAZymes profile in HF cross fed only finger millet straw (a common crop residue fed to ruminants in Karnataka, India), this study was undertaken with the key objectives of deciphering CAZymes diversity in HF cross cattle and to enumerate the composition of metabolically active, CAZyme-contributing microbiota that is involved in the hydrolysis of plant polysaccharides. A comparative analysis of the data obtained in our study and other published herbivore metagenomes was also performed to identify whether any unique CAZyme families exist in the HF cross rumen ecosystem.

## Materials and methods

### Experimental design and rumen sampling

Three fistulated Holstein–Friesian crossbred steers with an average body weight of 380 ± 15 kg were selected and maintained in individual stands for the feeding experiment at the Experimental Livestock Unit (ELU), National Institute of Animal Nutrition and Physiology, Bangalore, India. The animals were fed with finger millet straw, offered twice daily for a period of 21 days, at maintenance ration (ICAR 2013). The rumen contents were collected from all three animals prior to morning feeding on the last day of the experiment. Approximately 50 ml of rumen digesta samples were collected through the rumen fistula and immediately transported to the laboratory for further processing. Rumen digesta samples were mixed and strained through two layers of muslin cloth and immediately flash frozen in liquid nitrogen. Both liquid and solid portions of rumen digesta samples were then stored at −86 °C until further processing.

### Total DNA extraction from rumen digesta and quantification

The frozen rumen samples were thawed at room temperature and the solid rumen digesta samples were resuspended in phosphate buffered saline (Amresco, Solon, USA), for 2 h with vortexing to liberate the microbes adhering to feed particles, and mixed with the rumen fluid sample. The rumen fluid samples were then centrifuged at 4000 rpm for 5 min and the supernatant obtained was used further for the DNA extraction. In brief, the rumen fluid was centrifuged at 14,000 rpm for 10 min and the pelleted cells were resuspended in a mix of 800 µl of CTAB lysis buffer (2% CTAB, 1.4 M NaCl, 20 mM EDTA and 100 mM Tris–HCl), (Amresco, Solon, USA) and 0.2 g of glass beads (0.1 mm), (Biospec products Inc, Bartlesville, USA) and kept in a Mini bead beater (Biospec products Inc, Bartlesville, USA) for 3 min. 10 µl of 20 mg/ml proteinase K (Amresco, Solon, USA) and 10 mg/ml lysozyme (Amresco, Solon, USA) were added to the above mixture and incubated at 37 °C for 1 h. The tubes were then incubated at 70 °C for 30 min with intermittent mixing. An equal volume of Phenol:Chloroform:Isoamyl alcohol (25:24:1) (Amresco, Solon, USA) was added to the above lysate and mixed by inverting until a thick milky white emulsion was formed. After centrifugation at 14,000 rpm for 10 min, the supernatant was transferred to a fresh tube and total DNA was precipitated using 0.3 volumes of chilled ethanol (Merck, Kirkland, Canada). The precipitated DNA was then washed twice with 70% ethanol and the pellet was finally dried using a vacuum concentrator (Concentrator 5301) (Eppendorf, Hamburg, Germany). The quality of extracted genomic DNA was assessed by running it in 0.8% agarose gel electrophoresis for a single intact band, and A260/280 ratio was determined by Nanodrop 8000 (Thermo Fisher Scientific, Waltham, USA). Qubit 2.0 Fluorometer (Invitrogen, Carlsbad, USA) was used to measure the quantity of DNA.

### Metagenome library preparation and sequencing

The paired-end sequencing library was prepared using Illumina, Truseq Nano DNA LT Library Preparation Kit (Illumina, California, United States). Subsequently, 200 ng of genomic DNA was fragmented by Covaris (Covaris Inc, Massachusetts, USA) to generate a mean fragment distribution of 550 bp. The fragments were then subjected to end repair using end repair mix and indexing adapters were ligated to the ends of the DNA fragments. The ligated products were purified using SP beads supplied in the kit. The size-selected product was PCR amplified as described in the kit protocol. The amplified library was analyzed in Bioanalyzer 2100 (Agilent Technologies, California, USA) using a High Sensitivity DNA chip (Agilent Technologies, California, USA) as per the manufacturer’s instructions. The library was then loaded onto the Illumina MiSeq platform for cluster generation and subjected to paired-end sequencing.

### Metagenome assembly and bioinformatic analysis

De novo assembly of high quality data was accomplished using the CLC Genomics workbench 6.0 (Qiagen, USA) at default parameters (minimum contig length: 200, automatic word size: yes, perform scaffolding: yes, mismatch cost: 2, insertion cost: 3, deletion cost: 3, length fraction: 0.5, similarity fraction: 0.8). Bioinformatic analysis of the metadata was performed with Metagenome Rapid Annotation using Subsystem Technology (MG-RAST) (Meyer et al. 2008) server. The quality of the uploaded sequences was checked using MG-RAST quality filters and the sequences, which failed QC, were removed from further analysis. The metadata were functionally categorized via an RPS-BLAST comparison with the Subsystem database, (Overbeek et al. 2014), and KEGG databases (Kanehisa and Goto 2000).

### Carbohydrate-active enzyme annotation and taxonomic profiling

The fibrolytic gene encoding contigs from the metadata were identified and classified based on the carbohydrate-active enzymes database (Cantarel et al. 2009) (http://www.cazy.org) by the carbohydrate-active enzyme analysis toolkit (CAT) (Park et al. 2010) at an E value of 1 × 10^−5^. Putative plant cell wall polysaccharide-degrading enzymes belonging to different CAZy families were identified and classified based on sequence-based annotation. The CAZyme encoding contigs were analyzed manually for different classes of CAZymes: glycoside hydrolases (GHs), glycosyltransferases (GTs), carbohydrate esterases (CEs), carbohydrate-binding modules (CBMs), polysaccharide lyases (PLs), and auxiliary activities (AAs). The phylogenetic analysis of putative contigs encoding different CAZyme classes (24891 contigs) was performed in parallel to identify their microbial origin. The CAZyme encoding contigs from HF cross metagenome were uploaded on the MG-RAST server v 3.2 (Meyer et al. 2008) for phylogenetic analysis by the M5NR database using the BLASTX algorithm (Wilke et al. 2012) with a minimum identity of 60% and an E-value cutoff of 1 × 10^−5^. The CAZymes obtained in the present study were compared with other accessible metagenomic datasets, cow rumen (Hess et al. 2011), jersey cow (Wang et al. 2013), reindeer (Pope et al. 2012), macropod (Pope et al. 2010), and termite gut (Warnecke et al. 2007).

## Results

### Metagenome sequence data statistics and phylogenetic abundance

**Fig. 1:**
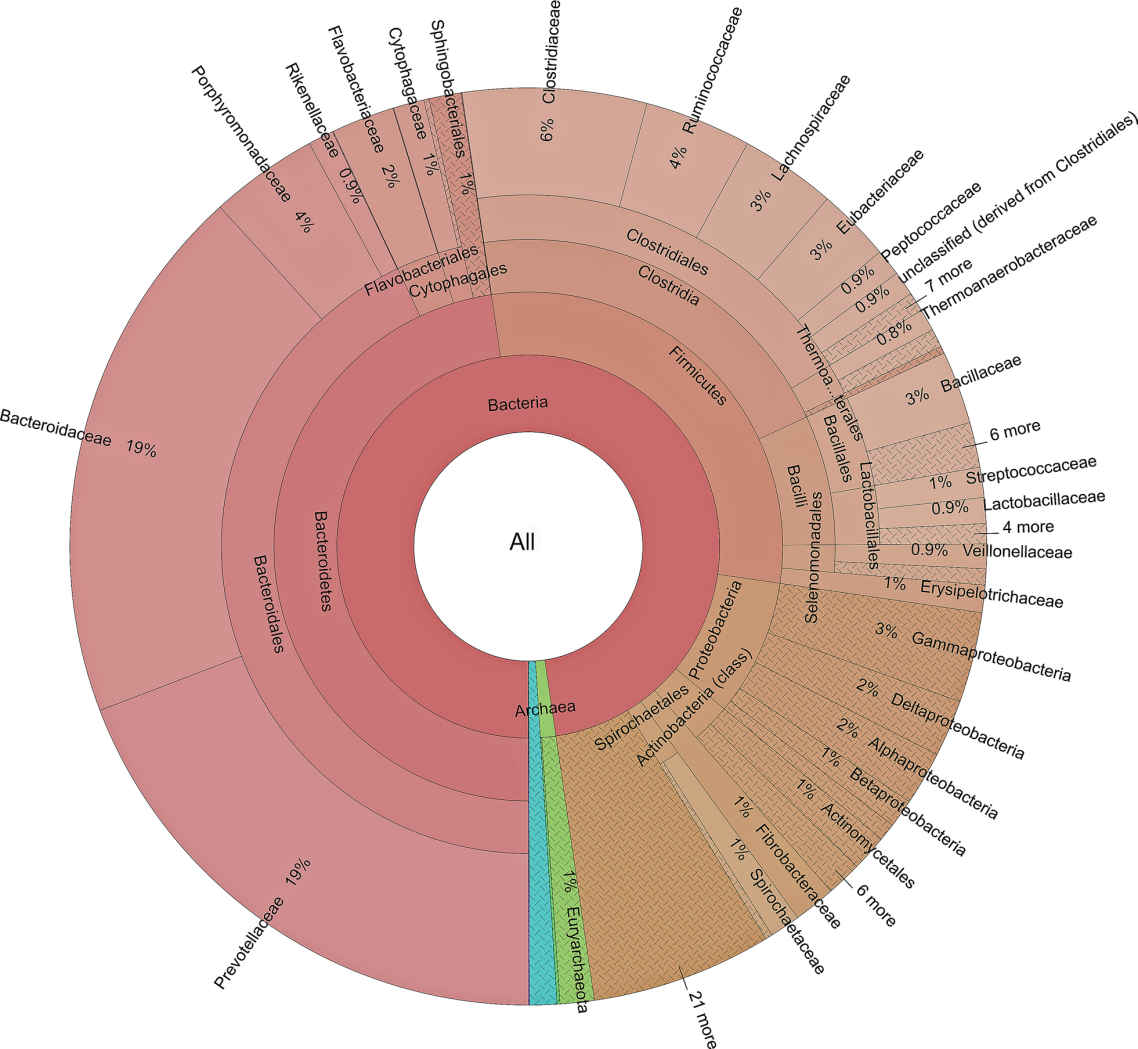
Krona chart illustrating the distribution of taxonomic domains indicating the percentages of reads with predicted proteins and ribosomal RNA genes annotated

The ultimate objective of our study was to elucidate the fibrolytic potential of the rumen microbial community in Indian crossbred (HF) cattle fed finger millet straw. The whole metagenome sequencing of the total DNA from cattle rumen digesta generated about 1.8 gigabases of raw sequences. De novo assembly of the raw sequencing reads after quality check (CLC Genomics Workbench 6.0) (Qiagen, USA) resulted in 171,594 contigs with an average length of 838 bp. The statistical elements of the assemblies were calculated by in-house perl scripts and the metagenomic data analysis statistics are given in Table 1. Contig-7574 was the largest contig with a length of 25,731 bps. In order to validate the contig assembly, 16 contigs (≥600 bp) from the glycoside hydrolase family were randomly selected and primers were designed to amplify the target gene fragment (Additional file 1: Table S1). Fifteen of the sixteen contigs were successfully amplified using at least one set of the primers and the sequences showed >99% identity to the assembled contigs. The metagenomic dataset was uploaded on the MGRAST server for further bioinformatic analysis. Phylogenetic analysis of metagenomic data at the domain level revealed that 97.5% of sequences binned to bacteria, 1.3% to archaea, and 0.9% to eukaryota (Fig. 1). At the genus level, the most predominant genera were *Prevotella*, *Bacteroides, Clostridium, Ruminococcus,* and *Parabacteroides*, representing more than 47% of the total sequences (Additional file 1: Table S2). The functional annotation using the SEED subsystem (Overbeek et al. 2014) has identified 73,886 predicted functions, out of which 17.6% corresponded to clustering-based subsystems, 9.7% with protein metabolism, and 9.9% to carbohydrates (Additional file 1: Figure S1). The key metabolic pathways and abundance of enzymes in HF cross rumen metadata were predicted using KEGG mapper (http://www.genome.jp/kegg/) and KEGG database (Kanehisa and Goto 2000) (Additional file 1: Figure S2; Table S3).

**Table 1:** Rumen metagenome data assembly analysis statistics by using in house perl scripts

Parameters	Number of sequences
Total number of bases uploaded	147,749,531
Total number of sequences uploaded	171,594
Mean sequence length bp uploaded	838 ± 481
Mean GC count uploaded	46 ± 10%
Artificial duplicate reads	14
Number of sequences failed QC	9310
Total number of bases post QC	125,833,189
Total number of sequences post QC	162,284
Mean sequence length post QC	775 ± 234
Mean GC count post QC	45 ± 10%
Predicted protein features	201,967
Predicted rRNA features	244
Identified protein features	97,723
Identified functional categories	58,691

### Mining of rumen metagenome for identification of fibrolytic enzymes

**Fig. 2:**
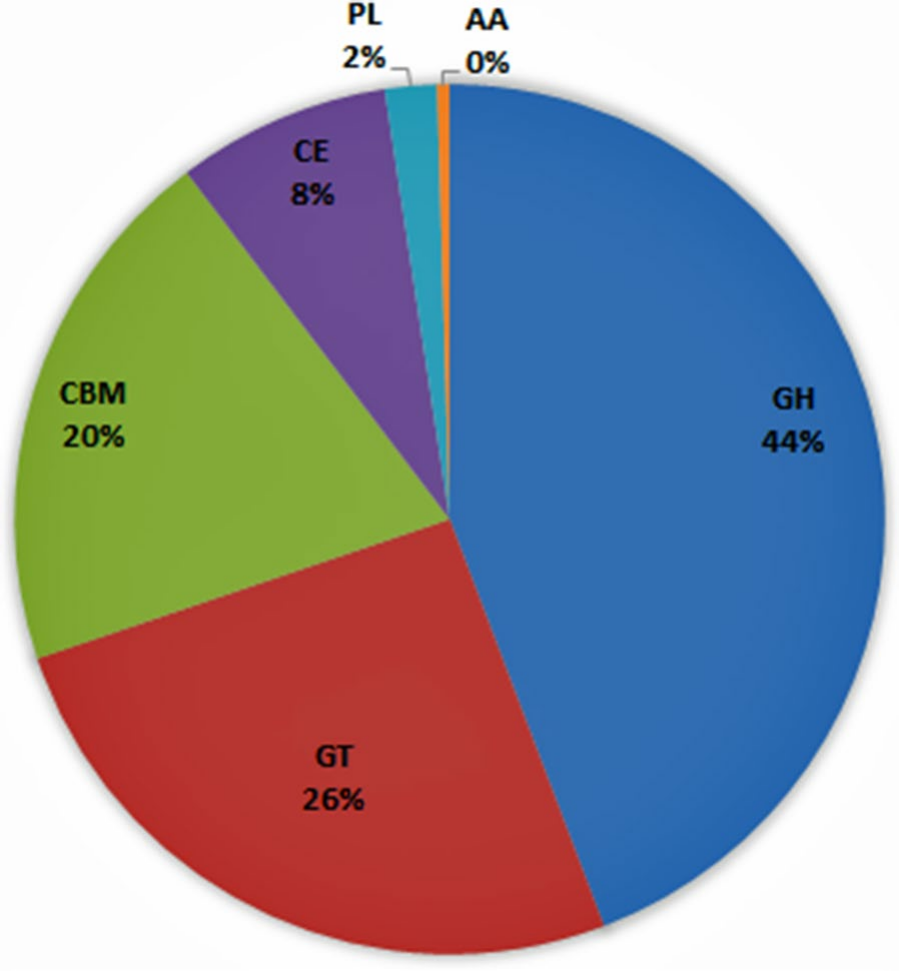
CAT analysis showing the distribution of different CAZyme classes across HF cross rumen metagenome. The *chart* shows the predominance CAZymes encoding putative glycoside hydrolases in the HF cross metagenome followed by glycosyltransferases and carbohydrate binding modules. *GH* glycoside hydrolase; *GT* glycosyltransferase; *CBM* carbohydrate-binding module; *PL* polysaccharide lyase; and *AA* auxiliary activities

Nucleotide sequence homology-based CAZymes annotation was performed for rumen metadata against the CAZy database (Cantarel et al. 2009) (http://www.cazy.org), using the CAZymes analysis Toolkit (CAT) (Park et al. 2010). CAT analysis using the assembled sequences (171,594 contigs) identified a total of 24,891 contigs (14.51% of total contigs) (Fig. 2) that had significant similarity with at least one of the reported CAZyme modules, spanning about 205 different CAZyme families (Additional file 1: Table S4). Approximately 13% of the contigs matching with different CAZyme classes could not be assigned to any of the protein families available in the CAZy database. CAT analysis revealed the number of contigs and respective Pfam domains of the members of different CAZyme classes that are mainly involved in the catalytic hydrolysis of plant cell wall polysaccharides inside cattle rumen.

The GH catalytic modules corresponding to 11,010 sequences were the most predominant and represented 96 different families altogether. The contigs encoding enzymes belonging to functional class GTs were the second most prominent (25.58% of CAZymes) group in HF cattle rumen and contained 6366 sequences from 40 different families. The enzymes from GT2, GT4, GT51, and GT28 families were present in larger proportion, representing 72.93% of the total GTs. CBM modules included 4945 sequences from 35 families. The CE1 family, encoding feruloyl esterases, which are essential for the solubilization of plant polymer lignin, was the principal CE family with 390 contigs in cattle rumen. There were 1975 contigs associated with thirteen CE families in the CAZy database, representing 7.93% of CAZymes. CAT analysis also detected the presence of fifteen families of PLs (1.93%) and six families of AAs (0.46%) in HF cross rumen metagenome (Additional file 1: Table S4).Out of 24,891 CAZyme encoding contigs, there were 2517 contigs (10.11%) that had two or more distinct CAZy domains. CBM domains were predominant among the multi-domain enzyme families accounting for about 1275 contigs (50.66%). Class glycoside hydrolases had 807 (32.06%) multi-domain contigs, largely associated with different carbohydrate-binding modules. CEs, GTs, and PLs represented 8.03, 5.44 and 3.77% of multi-domain enzymes, respectively, most of which were linked to CBMs.

A majority of the cattle rumen microbiome cellulases identified were classified as families GH5, GH6, GH9, GH44, GH45, and GH48. Gene fragments coding for putative cellulases belonging to families GH5 and GH9 accounted for 4% of the total GHs and were found to be the major families of cellulases present in cattle rumen. Likewise, 11 contigs encoding endoglucanases (GH45) and cellobiohydrolases (GH48) were also documented. Comparatively lesser number of cellulases degrading the main chains of plant cell wall components (10.67% of total GHs), were prevalent in HF rumen, while the enzymes involved in hydrolysis of side chains of plant polymers and oligosaccharides (GH1, GH2, GH3, GH29, GH35, GH38, GH39, GH42, GH43, and GH52), were in abundance and represented a majority of (60.11%) GHs. Putative exo-β-1, 4-glucanases (GH1 and GH3) and endo-β-1, 4-glucanases (GH5, and GH12) involved in the hydrolysis of β-1, 4-linked glucose residues from cellulose were represented by ~20 and ~4% of the total GHs, respectively. A large number of endo-hemicellulase degrading enzymes from GH8, GH10, GH11, GH12, GH26, GH28, and GH53 families were also identified and classified. CAT analysis also identified the presence of debranching enzymes belonging to family GH51 (α-l-arabino furanosidases), GH67 (α-glucuronidases), and GH 78 (α-l-rhamnosidases) that play a key role in depolymerization of hemicellulose. The enzymes effecting the hydrolysis of xylan main chains such as endo-1, 4-β-xylanase and endo-1, 3-β-xylanase were represented by GH10 and GH11 families. These accounted for about 2.09 and 0.3% of the total GHs, respectively (Table 2). Among the 96 GH families reported in this study, families GH3, GH2, and GH43 encoding oligosaccharide-degrading enzymes were most abundant and represented by 993, 927, and 739 contigs, respectively (Additional file 1: Table S4).

**Table 2:** Overview of the comparative analysis of putative carbohydrate-active enzymes belong to members of various GH families targeting plant structural polysaccharides identified in HF cross rumen with other herbivore metagenomes

GH family^a^	Major activity	HF cross	Jersey cow^b^	Cow^c^	Reindeer^d^	Termite^e^	Macropod^f^
Cellulases							
GH5	Cellulases	6.95	10.53	7.88	5.56	14.62	3.72
GH6	Endoglucanase	0.10	0.44	0.00	0.00	0.00	0.00
GH7	Endoglucanase	0.00	0.00	0.01	0.00	0.00	0.00
GH9	Endoglucanase	3.16	3.51	4.32	2.11	2.35	0.00
GH44	Endoglucanase	0.09	0.00	0.54	0.10	1.57	0.00
GH45	Endoglucanase	0.19	0.00	0.62	0.00	1.04	0.00
GH48	Cellobiohydrolases	0.19	0.00	0.02	0.10	0.00	0.00
Subtotal		10.67	14.47	13.39	7.87	19.58	3.72
Endo-hemicellulases							
GH8	Endoxylanase	1.14	0.00	1.79	0.68	1.31	0.37
GH10	Endo-1,4-β-xylanases	2.03	15.35	5.57	3.68	12.01	4.09
GH11	Xylanases	0.29	0.00	0.90	0.16	3.66	0.00
GH12	Xyloglucanases	0.53	0.44	0.00	0.00	0.00	0.00
GH26	β-Mannase and xylanases	1.16	0.44	2.00	2.97	3.92	1.86
GH28	Galacturonases	2.57	0.00	2.56	2.33	1.57	0.74
GH53	Endo-1,4-β-galactanases	10.19	7.89	2.62	2.42	3.13	3.35
Subtotal		17.92	24.12	15.45	12.23	25.59	10.41
Xylanoglucanases							
GH16	Xyloglucanases	2.53	0.00	2.62	2.25	0.26	1.49
GH74	Xyloglucanases	0.45	0.00	2.09	0.85	1.83	0.37
Subtotal		2.98	0.00	4.72	3.10	2.09	1.86
Debranching enzymes							
GH51	α-l-Arabinofuranosidases	0.48	0.44	6.79	9.46	4.70	4.46
GH54	α-l-Arabinofuranosidases	3.47	0.00	0.41	0.45	0.00	0.00
GH62	α-l-Arabinofuranosidases	0.00	0.00	0.01	0.00	0.00	0.00
GH67	α-Glucuronidases	0.98	0.00	0.65	1.43	2.61	1.86
GH78	α-l-Rhamnosidases	3.38	5.70	6.85	6.07	0.00	9.29
Subtotal		8.31	6.14	14.70	17.40	7.31	15.61
Oligosaccharide degrading enzymes							
GH1	β-Glucosidases	2.17	4.39	1.37	2.36	5.74	22.68
GH2	β-Galactosidases	15.99	7.02	7.80	13.88	6.01	8.92
GH3	β-Glucosidases	17.12	21.05	15.45	16.36	18.02	26.77
GH29	α-l-Fucosidases	2.45	1.32	5.10	5.19	0.00	0.74
GH35	β-Galactosidases	1.12	0.88	0.86	0.76	0.78	1.12
GH38	α-Mannosidases	0.79	0.44	1.48	2.25	2.87	1.12
GH39	β-Xylosidases	7.28	7.89	1.71	1.47	0.78	0.37
GH42	β-Galactosidases	0.45	0.00	2.03	1.84	6.27	2.97
GH43	Arabino/xylosidases	12.74	12.28	15.93	15.25	4.18	3.72
GH52	β-Xylosidases	0.00	0.00	0.01	0.04	0.78	0.00
Subtotal		60.11	55.26	51.75	59.40	45.43	68.40
Total		100.00	100.00	100.00	100.00	100.00	100.00
Metagenome size		1.8 Gb	0.28 Gb	268 Gb	0.30 Gb	0.062 Gb	0.054 Gb

Table lists the percentages of different glycoside hydrolase families targeting plant cell wall polysaccharides and their major activities. GHs are divided into different groups based on their activity and hydrolysis of plant polysaccharides. Enzymes belonging to oligosaccharide degrading cluster were observed as the predominant class representing a major proportion of GHs

^a^Based on CAZy data base (http://www.cazy.org)

^b^Wang et al. (2013) ^fed on Timothy grass hay^

^c^Hess et al. (2011) ^fed on switch grass^

^d^Pope et al. (2012) ^natural grazing on winter pastures, Norway^

^e^Warnecke et al. (2007) ^study on wood feeding termite^

^f^Pope et al. (2010) ^fed on Timothy cannary grass and commercial pellet^ ^mix^

### Microbial community analysis of CAZyme encoding contigs

**Fig. 3:**
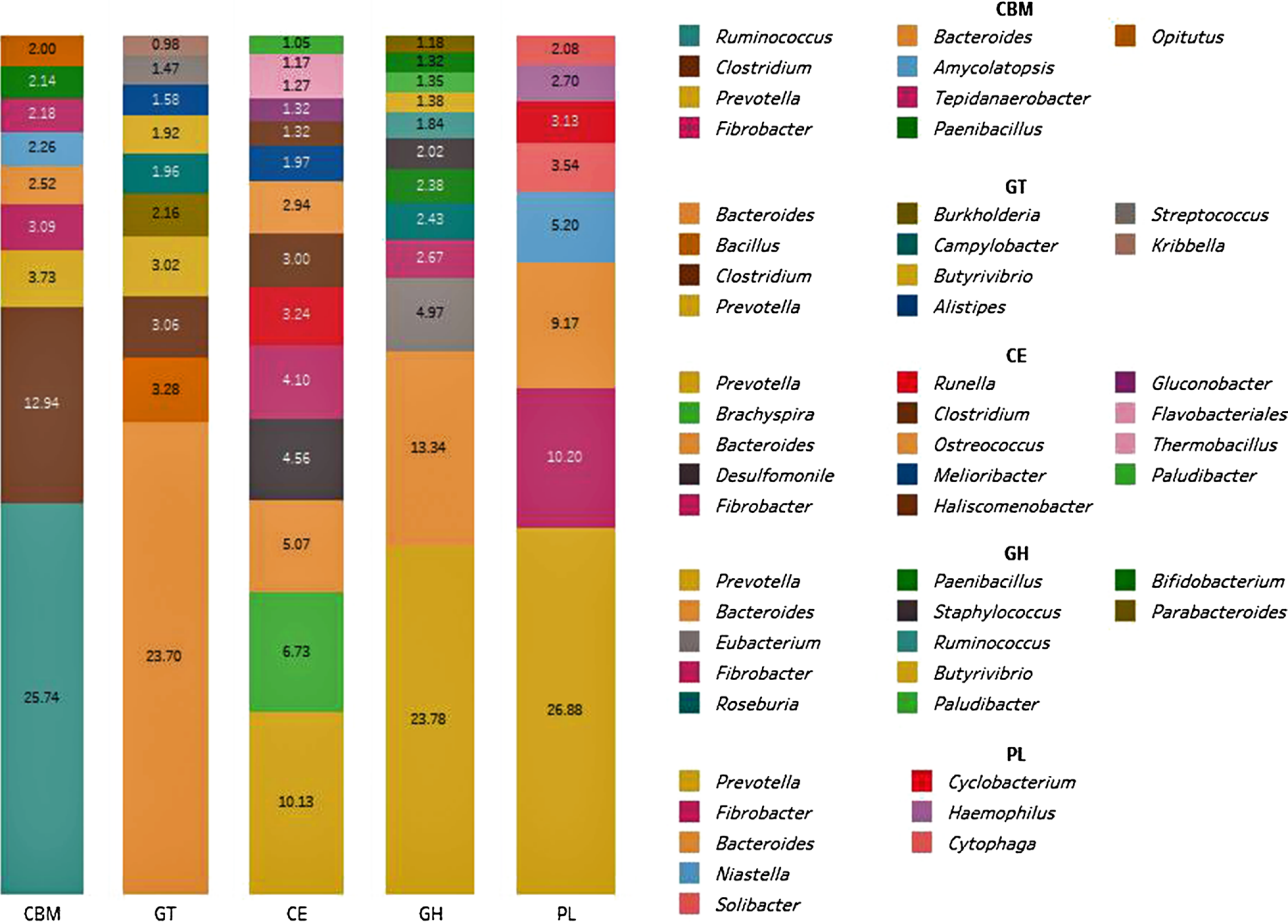
Taxonomic distribution of major CAZymes encoding contigs in HF cross rumen metagenome using M5NR database. The *bar chart* shows the percentages of contributions of CAZymes from the major microbial communities in cattle rumen. Bacteria belong to genus *Prevotella*, *Bacteroides, Fibrobacter, Clostridium,* and *Ruminococcus* were identified as the chief producers of CAZymes in cattle rumen ecosystem. *GH* glycoside hydrolase; *GT* glycosyltransferase; *CBM* carbohydrate-binding module; *PL* polysaccharide lyase; and *AA* auxiliary activities

To determine the phylogenetic origin of core microbial populations that significantly contribute CAZymes, all 24,891 CAZyme encoding regions, representing different classes of GHs, GTs, CBMs, CEs, PLs, and AAs, were analyzed separately. Phylogenetic analysis of CAZyme contigs on the MG-RAST server using the M5NR database revealed that the bacterial species belonging to genera *Prevotella, Bacteroides, Clostridium, Fibrobacter,* and *Ruminococcus* contributed a greater part of CAZyme encoding gene fragments in the HF cross rumen metagenome. Genera *Bacteroides* and *Prevotella* were the most dominant, producing a substantial amount of four major classes of CAZymes (GHs, GTs, CEs, and CBMs) accounting for about 44.63 and 36.1%, respectively. A significant proportion of GTs (23.7%), GHs (13.34%), and CEs (5.07%) were assigned to genus *Bacteroides* alone, whereas genus *Prevotella* was found to be the dominant contributor of GHs (19.22%), CEs (10.13%), and CBMs (3.73%). Genera *Fibrobacter* and *Clostridium* were found to encode three classes of CAZymes each: CBM, CE, GH and CBM, CE, GT, respectively. A major portion of CBMs were corresponding to members of genera *Ruminococcus* (25.74%) and *Clostridium* (12.94%) (Fig. 3).

## Discussion

### Rumen microbiota and CAZymes

Agricultural crop residues represent a significant proportion of renewable carbohydrate resources of energy for ruminants. The host as such cannot produce any enzymes that degrade the plant material, so they take advantage of the symbiotic association with rumen microflora to release the energy in form of carbohydrates and sugars from the recalcitrant plant polysaccharides. Rumen microbial populations are proven to play a vital role in plant fiber degradation in different ruminant species. A considerable variation in rumen microbiome has been observed across individual animals (Jami and Mizrahi 2012) and rumen microbial community structure also tends to vary significantly depending on the animal’s age and diet (Kittelmann and Janssen 2011; Li et al. 2012). The rumen microbial profile is also moderately influenced by breed, gender, and other ecological factors like grazing location (Jami and Mizrahi 2012). Nevertheless, most of the published studies on rumen microbial communities have identified the predominance of bacteria belonging to phyla *Bacteroidetes* and *Firmicutes*, irrespective of the change in diet, gender, breed, and other ecological factors (Kittelmann and Janssen 2011; Li et al. 2012; Petri et al. 2013; Stevenson and Weimer 2007; Thoetkiattikul et al. 2013). The results obtained in our studies confirm the earlier reports where the bacteria belonging to phyla *Bacteroidetes* and *Firmicutes* are the major contributors of different classes of CAZymes in the cattle rumen ecosystem. This result may indicate that the bacterial populations inhabiting the cattle rumen are largely similar to those of other ruminants in terms of *Bacteroidetes* and *Firmicutes* populations. The enzymes produced by these microbial communities are reported to have the potential to digest plant polymers like xylan, pectin, and starch (Stevenson and Weimer 2007). Genus *Prevotella* which has been established to account for a significant part of genetic and metabolic diversity of microbial communities in ruminants (Purushe et al. 2010). This genera was found to contribute a substantial proportion of CAZymes (>36%) in our study, when the animals were fed with only finger millet straw, that is rich in fiber. *Prevotella* has already been reported to play a vital part when there was a shift from a high-calorie diet to high-fiber diet (Jami et al. 2013). Microbes belonging to both genera *Bacteroides* and *Prevotella* were found to possess a large repertoire of CAZymes; *Bacteroides* were the chief manufacturers of CAZyme class GTs, whereas *Prevotella* were the principal contributors of GHs in cattle rumen. The natural capability of rumen microbiota to produce an array of potential enzymes that hydrolyze the rigid lignocellulose biomass has been successfully employed to treat different systems like agricultural residues and straw waste (Hu et al. 2007; Barnes and Keller 2004). The application of different artificial rumen systems for organic waste conversion (Yue et al. 2013) was also being extensively studied. Plant biomass containing cellulose and hemicellulose on earth is considered to be one of the largest sources of fermentable sugars and energy that could be utilized for various industrial applications, like ethanol production (Jorgensen et al. 2007). Despite the large-scale availability of these plant polysaccharides, the major challenge for accessing these fermentable sugars is the presence of a highly resilient aromatic compound called lignin (Alvira et al. 2010). However, the ruminants with their highly complex efficient microbial communities can produce a broad range of fibrolytic enzymes that can facilitate the hydrolysis of lignocellulose biomass (Hess et al. 2011). However, it should be noted that the NGS-based Illumina-MiSeq sequencing platform employed in this study cannot be used for the quantitative interpretation of the rumen microbial community structure and size due to the bias inherent with the PCR amplification and sequencing of 16s rRNA gene, as it may result in misinterpretation of active community members (Schloss et al. 2011).

### Cattle rumen has highly diverse and complex CAZymes

The CAZy database (http://www.cazy.org) exclusively deals with the diverse group of enzymes that actively contribute to synthesis and degradation of complex carbohydrates and glycoconjugate. The CAZy database provides manually curated information for all CAZyme families: glycoside hydrolases, glycosyltransferases, polysaccharide lyases, carbohydrate esterases, carbohydrate-binding modules, and auxiliary activities; it now allows one to examine all known families and enzymes involved in cellulolysis, hemicellulolysis, and pectienolysis (Cantarel et al. 2009). Enzymes coding for GH families are highly abundant in most of the genomes and they account for about 50% of the enzymes classified in the CAZy database. Among all major classes of CAZymes that have been reported till date, glycoside hydrolases were observed to be the most predominant and diverse group of catalytic enzymes involved in the hydrolysis of plant polymers in Indian crossbred cattle rumen metagenome.

GHs representing glycosidases and transglycosidases are responsible for the hydrolysis of the glycosidic bonds linking two or more carbohydrates or a carbohydrate and a non-carbohydrate moiety accounting for about 6.5% of the total sequences. The total number of contigs obtained in each CAZyme family gives an indication of the abundance of that particular group of enzymes in the HF cross cattle rumen. Out of 135 CAZy families that are reported in the CAZy database till date, 96 families were represented by cattle rumen metagenome. Patel et al. (2014) reported 72 families of GHs from the buffalo rumen ecosystem. The substantially large number of enzymes from the GH family (96 families) in our study could be either due to the difference in NGS sequencing platform or the modifications in the analysis pipe line. Considerably higher number of GH families obtained in our study also indicates that HF cross rumen might have a more intricate process of lignocellulose breakdown than other reported rumen metagenomes.

In parallel to previous reports (Wang et al. 2013; Stevenson and Weimer 2007; Pope et al. 2012), genes encoding cellulases belonging to families GH5 and GH9 were present in higher proportion than other cellulase coding families. Dai et al. (2012) reported the exoglucanase activity of enzymes corresponding to the GH5 family which is known for endoglucanase, mannanase and endo-xylanase activities. The large number of putative cellulase genes identified in the GH5 family (403 contigs) in our study suggests that cattle rumen microbes may have novel strategies for degradation of plant polysaccharides present in the feed. Previous studies on rumen metagenomes have not reported any contigs assigned to the GH6 family, other than one contig from the fosmid library of jersey cow rumen metagenome (Wang et al. 2013). Conversely, Indian crossbred cattle rumen microbiome was found to exhibit a marginally higher amount of endoglucanases corresponding to the GH6 family (6 contigs).Consistent with earlier herbivore-related metagenome and metatranscriptome studies (Brulc et al. 2009; Hess et al. 2011; Pope et al. 2012; Warnecke et al. 2007; Qi et al. 2011) no contigs associated with the GH7 cellulase family were found. Consequently, the scanty representation (11 contigs) of enzymes belonging to the GH48 family displays the possibilities of the non-cellulosomal mode of plant cell wall hydrolysis in cattle rumen. Additionally, the contigs which were affiliated to diverse CAZyme modules (~13%), but could not be classified under any of the known or reported Pfam domains, indicated that the cattle rumen microbiome produces a large number of unique CAZymes which are meagerly characterized. Complete depolymerization of plant polysaccharides requires collective activities of different groups of enzymes other than GHs and these include GTs, CBMs, CEs, PLs, and AAs. GTs, reported to be involved in the catalysis of glycosidic bonds to form disaccharides, oligosaccharides, and polysaccharides from phospho-activated sugar donors (Coutinho et al. 2003; Lairson et al. 2008), were the second most abundant CAZy family HF rumen (~4% of total contigs). Out of the forty GT families identified in cattle rumen metagenome, enzymes belonging to families GT2 and GT4 (cellulose synthase, chitin synthase, α-glucosyltransferase, etc.) represented a significant proportion (>62%) of the total GTs. CBMs which have no reported enzymatic activity on their own, but can potentiate the activities of all other CAZymes (GHs, CEs, and auxiliary enzymes) or act as an appendix module of CAZymes (Tomme et al. 1995; Boraston et al. 2004), accounting for 2.9% of contigs.

In conclusion, high throughput sequencing-based whole metagenomic approach was used to investigate the natural biomass-converting microbial communities in the cattle rumen ecosystem. Comprehensive analysis of metagenome data strongly indicates that the complex digestive system of Indian crossbred cattle possesses a very high degree of a deeply branched and extremely diverse group of enzymes of microbial origin which have the ability to degrade the recalcitrant lignocellulose plant biomass. The CAZymes corresponding to class glycoside hydrolases were found to be the most abundant and diverse group of CAZymes in cattle rumen. Bacteria belonging to genera *Prevotella, Bacteroides, Clostridium, Fibrobacter,* and *Ruminococcus* were identified as the key contributors of CAZymes inhabiting the cattle rumen. This study provides a substantially expanded catalogue of enzymes that participate in the deconstruction of plant cellulosic biomass in Indian cattle rumen, which provides an opportunity to improve ruminant nutrition and also to develop proficient fermentation systems for bioconversion of plant biomass into biofuels.

## Additional file

**Additional file 1.** Additional tables and figures.

## Abbreviations

CAZymes: carbohydrate active enzymes
HTS: high throughput sequencing
HF: Holstein Friesian
ELU: experimental livestock unit
CTAB: cetyl trimethylammonium bromide
MG-RAST: Metagenome Rapid Annotation using Subsystem Technology
QC: quality control
RPS BLAST: reversed position specific basic local alignment search tool
KEGG: Kyoto Encyclopedia of Genes and Genomes
CAT: carbohydrate-active enzyme analysis toolkit
GHs: glycoside hydrolases
GTs: glycosyl transferases
CEs: carbohydrate esterases
CBMs: carbohydrate-binding modules
PLs: polysaccharide lyases
AAs: auxiliary activities
