# Supplementary material for: Metagenomic insights into the rumen microbial fibrolytic enzymes in Indian crossbred cattle fed finger millet straw
Source: AMB Express. 2017 Jan 3;7:13. doi: 10.1186/s13568-016-0310-0 (PMC5209320; doi:10.1186/s13568-016-0310-0)

Journal Name: AMB Express

Title: Metagenomic Insights into the Rumen Microbial Fibrolytic Enzymes in Indian Crossbred Cattle Fed Finger Millet Straw

Authors: V. Lyju Jose[1, 2], Thulasi Appoothy[1]*, Ravi P More[3] and A Sha Arun[4]

[1] Rumen Microbiology Laboratory, Animal nutrition Division, National Institute of Animal Nutrition and Physiology, Bangalore, India,

[2] PhD Scholar, Department of Biotechnology, Jain University, Bangalore, India

[3] Bioinformatics Laboratory, Division of Molecular Entomology, National Bureau of Agricultural Insect Resources, Bangalore, India.

[4] Wildlife SOS, Bannerghatta Bear Rescue Centre, Bangalore, India.

***Correspondence:**

Dr. Thulasi Appoothy, thulasi.appoothy@gmail.com, Ph: 080 25711304, Fax: 080 25711420

Supplementary Table S1. Primers designed for validating contigs encoding lignocellulolytic enzymes from HF cross rumen metagenomic data

S No	Contig number		Length	Tm	GC%	Sequence	Product size bp
1	contig_3500	FP	20	59.9	55	GCCAAGGTCAAGAAGACCAG CAACGAGTACCCCAACGTCT	220
		RP	20	59.9	50	TCGTCGTGCGAATAGATGAG TGCATCGGTGAGGTTGTAGA	
2	contig_320985	FP	20	60.1	50	CCGTCGTGGAATTCTTCAGT	207
		RP	20	59.9	55	CGACGAACTCCTTCTCCTTG	
3	contig_450358	FP	20	60.1	51	ATGCGGTTCAAGGAAGTGAC	215
		RP	20	60	55	GAGCCCGATATTCTCCACAA ATGCAAGAGGTCATCGGTTC	
4	contig_80122	FP	20	59.8	48	AGCCTTTCGACTGGCAATTA	250
5		RP	20	59.9	50	TTGAAATGCATGTCGGTGAT	
6	contig_96204	FP	20	59.2	50	TCAACTTTGCCCCCTGTATC	325
		RP	20	59.9	50	GTGATCCAAAGCAGGGATGT GTGATCCAAAGCAGGGATGT	
7	contig_118682	FP	20	59.4	48	ATCAGTCCGCAAAAACCATC	564
		RP	20	60	50	AGGAGCTGAAGCTGGACGTA	
8	contig_142716	FP	20	60.9	60	CGCAGAGGTAGGTGACGAAG CGCAGAGGTAGGTGACGAA	370
		RP	20	60.7	50	AACATGATCTGGGACCATCG	
9	contig_295107	FP	20	60.8	50	ACTCATTGAAGACGGCATCC	338
		RP	20	59.8	50	GGAGCATAAGGTGCTGAAGG	
10	contig_318368	FP	20	59.8	50	ACGGATGTGGAGATCGAAAC	515
		RP	20	60.2	50	CTTTCTTCAGCGCTTTCAGG CCTTTCTTCAGCGCTTTCAG	
11	contig_498267	FP	20	59.2	45	AAGGGCAAGCATACCATTTG	376
		RP	20	60.1	45	CATCAAAATGTGGAGCGATG	
12	contig_62300	FP	20	59.1	50	CGGAAGTACTGGCCCATAAA	564
		RP	20	60.1	55	CACATCACACAGGGTTCGAC	
13	contig_403730	FP	20	60.1	50	CGAATCGTCGTTTCCTTGAG	510
		RP	20	59.9	50	ATATCACACCACTGGCCACA GCGGCAGTCGTCTTCAGTAT	
14	contig_198814	FP	20	61.1	50	ACCTCCACAAAACCAGTTGC	594
		RP	20	60.8	52	GATATGCACAGGGCGAGATT	
15	contig_309249	FP	20	59.8	50	GTTTACCATGCGGTTCACCT	471
		RP	20	60.2	55	GTGGAAGGCTCCACGATCTA	
16	contig_384915	FP	20	59.9	55	GAGGATGAGCAGGTTGAAGC	448
		RP	20	59.7	50	GTGGTATCCCGTTTTCCAGA	

Supplementary Table S2. Taxonomic distribution of cattle rumen metagenomic data at genus level showing the major genera along with the number of reads assigned to each genera

**Genus Name**	**Abundance**
Prevotella	37757
Bacteroides	37534
Clostridium	12149
Ruminococcus	5124
Parabacteroides	5092
Eubacterium	4883
Bacillus	3615
Fibrobacter	2905
Butyrivibrio	2631
Streptococcus	1879
Alistipes	1775
Lactobacillus	1714
Blautia	1611
unclassified (derived from *Erysipelotrichaceae)*	1462
Roseburia	1396
Porphyromonas	1287
Treponema	1247
unclassified (derived from *Peptostreptococcaceae)*	1136
Paludibacter	1102
unclassified (derived from *Clostridiales)*	1020
Paenibacillus	1004
Faecalibacterium	981
Flavobacterium	963
Desulfitobacterium	959
Coprococcus	950
Victivallis	910
Caldicellulosiruptor	875
Geobacillus	873
Bifidobacterium	799
Fusobacterium	775
Geobacter	768
unclassified (derived from *Lachnospiraceae)*	761
Acidaminococcus	737
Staphylococcus	727
Capnocytophaga	724
Pedobacter	708
Desulfovibrio	706
Listeria	701
Spirosoma	680
Spirochaeta	678
Chitinophaga	669
Enterococcus	613
Thermoanaerobacter	608
Alkaliphilus	604
Selenomonas	594
Chlorobium	584
Dyadobacter	580

unclassified (derived from *Bacteria)*	556
Desulfotomaculum	540
Dorea	510
Mycoplasma	509
Holdemania	508
Vibrio	504
Methanobrevibacter	495
Coprobacillus	470
Cytophaga	461
Shewanella	455
Slackia	455
Pseudoflavonifractor	452
Pseudomonas	447
Streptomyces	443
unclassified (derived from *Bacteroidetes)*	442
Campylobacter	422
Veillonella	422
Methanosarcina	416
Abiotrophia	414
Phascolarctobacterium	380
Subdoligranulum	379
Caldanaerobacter	376
Burkholderia	372
Sphingobacterium	371
Oribacterium	369
Leeuwenhoekiella	358
Synechococcus	356
Zunongwangia	348
Acetivibrio	346
unclassified (derived from *Ruminococcaceae)*	338
Polaribacter	330
Catenibacterium	324
Acholeplasma	315
Cellulosilyticum	305
Anaerotruncus	304
unclassified (derived from unclassified sequences)	298
Carboxydothermus	296
Escherichia	293
Thermotoga	293
Brachyspira	287
Candidatus Solibacter	281
Maribacter	276
Ethanoligenens	275
Gramella	269
Pelobacter	268
Mucilaginibacter	255
Dictyoglomus	253
Helicobacter	253
Magnetospirillum	249
Leptotrichia	245

Eggerthella	242
Mitsuokella	240
Pelodictyon	238
Anaeromyxobacter	237
Thermoanaerobacterium	236
Anaerostipes	235
Opitutus	228
Turicibacter	226
Microscilla	224
Atopobium	223
Corynebacterium	220
Mycobacterium	220
Neisseria	220
Anoxybacillus	219
Haemophilus	216
Marivirga	213
Lactococcus	212
Dokdonia	210
Shuttleworthia	210
unclassified (derived from *Flavobacteriales)*	210
Oceanobacillus	209
Xanthomonas	208
Halothermothrix	207
Leptospira	207
Borrelia	199
Moorella	197
Actinobacillus	191
Collinsella	191
Leadbetterella	191
Desulfuromonas	189
Propionibacterium	188
Syntrophomonas	188
Chlorobaculum	186
Trichomonas	186
Anaerococcus	185
Elusimicrobium	185
Rhodopirellula	183
Exiguobacterium	182
Thermincola	181
Akkermansia	179
Syntrophobacter	178
Cyanothece	177
Peptoniphilus	177
Planctomyces	173
Francisella	165
Yersinia	163
Kordia	159
Rhizobium	159
unclassified (derived from *Flavobacteriia)*	159
Lentisphaera	158

Alicyclobacillus	156
Deinococcus	155
Dialister	155
Brevibacillus	153
Rickettsia	152
Algoriphagus	151
Lysinibacillus	151
Finegoldia	148
Rhodopseudomonas	148
Ilyobacter	147
Acinetobacter	146
Thermosinus	145
Robiginitalea	144
Desulfococcus	142
Halanaerobium	141
Rhodospirillum	140
Rhodothermus	140
Prochlorococcus	139
Solobacterium	138
Bradyrhizobium	137
Pelotomaculum	137
Symbiobacterium	137
Methanococcus	136
Thermosipho	136
Anaerofustis	135
Methylobacterium	134
Pyramidobacter	133
unclassified (derived from *Siphoviridae)*	133
Desulfatibacillum	131
Croceibacter	126
Candidatus Koribacter	125
Dehalococcoides	125
Leuconostoc	125
Acidovorax	123
Diplosphaera	123
Sorangium	123
Chryseobacterium	122
Desulfotalea	122
Pedosphaera	122
Bulleidia	121
Chloroflexus	120
unclassified (derived from *Elusimicrobia)*	119
Olsenella	118
Thermosediminibacter	118
Chloroherpeton	116
Peptostreptococcus	116
Chthoniobacter	114
Fervidobacterium	114
Pseudoalteromonas	114
Psychroflexus	114

Aeromonas	113
Erysipelothrix	111
Nostoc	111
Salmonella	111
unclassified (derived from *Deltaproteobacteria)*	111
Cryptobacterium	110
Enterobacter	110
Photobacterium	110
Sebaldella	109
Roseiflexus	105
Verrucomicrobium	105
Candidatus Phytoplasma	104
Desulfobacterium	104
Myxococcus	104
Caulobacter	103
Megasphaera	103
Dethiosulfovibrio	102
Methanothermobacter	102
Petrotoga	102
Methanocaldococcus	101
Saccharophagus	101
Syntrophus	101
Heliobacterium	100
Sulfurihydrogenibium	100

Supplementary Table S3. List showing the names and abundance of enzymes identified in cattle rumen metadata using KEGG database

EC number	Enzyme Name	Abundance
3.6.4.12	DNA helicase	589
2.7.7.7	DNA-directed DNA polymerase	553
2.7.7.6	DNA-directed RNA polymerase	371
3.2.1.21	beta-glucosidase	358
3.6.3.14	H+-transporting two-sector ATPase	311
3.2.1.23	beta-galactosidase	235
6.3.5.5	Carbamoyl-phosphate synthase (glutamine-hydrolysing)	168
2.7.6.5	GTP diphosphokinase	162
2.1.1.37	DNA (cytosine-5-)-methyltransferase	155
1.17.4.2	ribonucleoside-triphosphate reductase	155
2.2.1.1	transketolase	150
6.1.1.20	Phenylalanine-tRNA ligase	142
6.2.1.3	long-chain-fatty-acid-CoA ligase	142
1.6.5.3	NADH:ubiquinone reductase (H+-translocating)	141
2.4.1.1	glycogen phosphorylase	137
6.5.1.2	DNA ligase (NAD+)	134
6.1.1.5	Isoleucine-tRNA ligase	130
5.4.2.1	phosphoglycerate mutase --> transferred. Now recognized as two separate enzymes EC 5.4.2.11, phosphoglycerate mutase (2,3-diphosphoglycerate-dependent) and EC 5.4.2.12, phosphoglycerate mutase (2,3-diphosphoglycerate-independent)	118
6.3.1.2	Glutamate-ammonia ligase	117
6.1.1.10	Methionine-tRNA ligase	112
3.4.21.53	Endopeptidase La	105
2.7.1.48	uridine kinase	105
5.4.2.8	phosphomannomutase	103
6.1.1.7	Alanine-tRNA ligase	103
1.1.1.100	3-oxoacyl-[acyl-carrier-protein] reductase	95
3.2.1.22	alpha-galactosidase	94
6.1.1.3	Threonine-tRNA ligase	93
6.1.1.12	Aspartate-tRNA ligase	89
6.1.1.4	Leucine-tRNA ligase	89
2.5.1.49	O-acetylhomoserine aminocarboxypropyltransferase	89
3.6.1.1	inorganic diphosphatase	88
2.1.1.13	methionine synthase	86
3.1.26.4	ribonuclease H	86
6.1.1.9	Valine-tRNA ligase	86
3.2.1.52	beta-N-acetylhexosaminidase	86
5.1.3.2	UDP-glucose 4-epimerase	85
6.1.1.6	Lysine-tRNA ligase	82
6.1.1.19	Arginine-tRNA ligase	82
2.5.1.75	tRNA dimethylallyltransferase	82
6.1.1.17	Glutamate-tRNA ligase	81
6.3.5.3	phosphoribosylformylglycinamidine synthase	80
6.1.1.21	Histidine-tRNA ligase	80
2.7.7.13	mannose-1-phosphate guanylyltransferase	79

2.4.2.14	amidophosphoribosyltransferase	79
2.7.1.11	6-phosphofructokinase	78
2.4.1.25	4-alpha-glucanotransferase	78
2.7.7.24	glucose-1-phosphate thymidylyltransferase	77
6.2.1.30	phenylacetate-CoA ligase	76
2.4.1.18	1,4-alpha-glucan branching enzyme	76
1.2.7.3	2-oxoglutarate synthase	75
3.4.14.5	dipeptidyl-peptidase IV	75
6.1.1.2	Tryptophan-tRNA ligase	75
2.7.13.3	histidine kinase	73
1.1.1.22	UDP-glucose 6-dehydrogenase	73
6.3.2.13	UDP-N-acetylmuramoyl-L-alanyl-D-glutamate-2,6-diaminopimelate ligase	73
6.1.1.15	Proline-tRNA ligase	72
3.2.1.51	alpha-L-fucosidase	72
2.7.7.8	polyribonucleotide nucleotidyltransferase	72
6.1.1.11	Serine-tRNA ligase	71
2.7.1.2	glucokinase	70
4.2.1.20	tryptophan synthase	70
6.3.2.10	UDP-N-acetylmuramoyl-tripeptide-D-alanyl-D-alanine ligase	69
2.4.1.21	starch synthase (glycosyl-transferring)	67
2.3.1.54	formate C-acetyltransferase	67
1.17.7.1	(E)-4-hydroxy-3-methylbut-2-enyl-diphosphate synthase	67
6.3.4.4	Adenylosuccinate synthase	67
2.7.4.16	thiamine-phosphate kinase	66
6.3.5.4	Asparagine synthase (glutamine-hydrolysing)	66
2.8.1.7	cysteine desulfurase	65
2.6.1.1	aspartate transaminase	65
5.1.3.14	UDP-N-acetylglucosamine 2-epimerase (non-hydrolysing)	65
2.7.6.1	ribose-phosphate diphosphokinase	64
3.2.1.55	non-reducing end alpha-L-arabinofuranosidase	64
2.7.1.6	galactokinase	63
4.2.3.1	threonine synthase	63
2.3.1.179	beta-ketoacyl-[acyl-carrier-protein] synthase II	63
5.3.1.9	Glucose-6-phosphate isomerase	62
4.2.99.18	DNA-(apurinic or apyrimidinic site) lyase	62
2.2.1.6	acetolactate synthase	62
6.2.1.1	acetate-CoA ligase	62
4.1.1.20	Diaminopimelate decarboxylase	62
6.3.2.4	D-Alanine-D-alanine ligase	61
2.3.1.30	serine O-acetyltransferase	61
2.7.2.1	acetate kinase	61
1.1.1.95	phosphoglycerate dehydrogenase	60
1.8.1.9	thioredoxin-disulfide reductase	60
2.4.1.227	undecaprenyldiphospho-muramoylpentapeptide beta-N-acetylglucosaminyltransferase	60
2.5.1.47	cysteine synthase	60
1.4.4.2	glycine dehydrogenase (decarboxylating)	60
2.4.2.9	uracil phosphoribosyltransferase	60
6.3.4.3	formate-tetrahydrofolate ligase	60
4.4.1.8	cystathionine beta-lyase	59
4.3.3.7	4-hydroxy-tetrahydrodipicolinate synthase	59

6.3.2.8	UDP-N-acetylmuramate-L-alanine ligase	58
1.1.1.205	IMP dehydrogenase	58
6.1.1.14	glycine-tRNA ligase	58
2.5.1.6	methionine adenosyltransferase	58
4.3.2.1	argininosuccinate lyase	57
3.4.16.4	serine-type D-Ala-D-Ala carboxypeptidase	57
3.1.11.5	exodeoxyribonuclease V	57
5.1.3.13	dTDP-4-dehydrorhamnose 3,5-epimerase	57
2.7.9.1	pyruvate, phosphate dikinase	57
2.5.1.7	UDP-N-acetylglucosamine 1-carboxyvinyltransferase	57
2.7.2.4	aspartate kinase	56
2.6.1.9	histidinol-phosphate transaminase	56
2.4.2.1	purine-nucleoside phosphorylase	56
4.1.1.3	Oxaloacetate decarboxylase	55
3.4.21.92	Endopeptidase Clp	55
6.1.1.1	Tyrosine-tRNA ligase	54
1.1.1.267	1-deoxy-D-xylulose-5-phosphate reductoisomerase	54
1.3.99.22	coproporphyrinogen dehydrogenase	54
6.3.2.9	UDP-N-acetylmuramoyl-L-alanine-D-glutamate ligase	53
3.2.1.20	alpha-glucosidase	53
6.3.5.2	GMP synthase (glutamine-hydrolysing)	53
1.2.1.41	glutamate-5-semialdehyde dehydrogenase	53
2.1.2.9	methionyl-tRNA formyltransferase	53
3.5.99.6	glucosamine-6-phosphate deaminase	53
4.3.2.2	adenylosuccinate lyase	53
4.2.1.46	dTDP-glucose 4,6-dehydratase	52
4.2.3.5	chorismate synthase	52
2.4.1.129	peptidoglycan glycosyltransferase	51
3.5.2.3	dihydroorotase	51
5.1.3.1	ribulose-phosphate 3-epimerase	51
2.6.1.16	glutamine-fructose-6-phosphate transaminase (isomerizing)	51
1.3.99.1	succinate dehydrogenase	51
3.4.21.89	Signal peptidase I	51
6.1.1.16	Cysteine-tRNA ligase	51
5.1.3.3	Aldose 1-epimerase	50
1.2.1.11	aspartate-semialdehyde dehydrogenase	50
2.5.1.31	ditrans,polycis-undecaprenyl-diphosphate synthase [(2E,6E)-farnesyl-diphosphate specific]	50
3.6.4.13	RNA helicase	50
4.2.1.2	fumarate hydratase	49
2.7.7.27	glucose-1-phosphate adenylyltransferase	49
2.3.1.180	beta-ketoacyl-[acyl-carrier-protein] synthase III	49
2.3.1.15	glycerol-3-phosphate 1-O-acyltransferase	49
3.1.5.1	dGTPase	49
1.1.1.158	UDP-N-acetylmuramate dehydrogenase --> transferred, now EC 1.3.1.98	49
6.3.4.2	CTP synthase (glutamine hydrolysing)	48
2.7.8.13	phospho-N-acetylmuramoyl-pentapeptide-transferase	48
6.1.1.18	Glutamine-tRNA ligase	48
4.1.2.13	fructose-bisphosphate aldolase	48
2.1.1.45	thymidylate synthase	48
4.1.3.27	anthranilate synthase	47

4.2.3.4	3-dehydroquinate synthase	47
3.1.11.6	exodeoxyribonuclease VII	47
1.1.1.94	glycerol-3-phosphate dehydrogenase [NAD(P)+]	47
2.1.2.2	phosphoribosylglycinamide formyltransferase	46
3.6.1.23	dUTP diphosphatase	46
2.7.4.8	guanylate kinase	46
2.6.1.83	LL-diaminopimelate aminotransferase	46
2.7.2.3	phosphoglycerate kinase	46
6.3.5.1	NAD+ synthase (glutamine-hydrolysing)	45
1.3.98.1	dihydroorotate dehydrogenase (fumarate)	45
3.1.3.5	5'-nucleotidase	44
1.1.1.23	histidinol dehydrogenase	44
2.7.1.4	fructokinase	44
2.3.1.41	beta-ketoacyl-[acyl-carrier-protein] synthase I	43
1.4.1.4	glutamate dehydrogenase (NADP+)	43
1.3.1.26	dihydrodipicolinate reductase --> transferred, now EC 1.17.1.8	42
2.3.1.47	8-amino-7-oxononanoate synthase	42
6.3.4.13	phosphoribosylamine-glycine ligase	42
5.1.1.7	diaminopimelate epimerase	42
4.1.2.4	deoxyribose-phosphate aldolase	42
2.3.1.39	[acyl-carrier-protein] S-malonyltransferase	41
4.2.1.59	3-hydroxyacyl-[acyl-carrier-protein] dehydratase	41
2.7.4.3	adenylate kinase	41
2.7.1.17	xylulokinase	41
5.3.1.6	Ribose-5-phosphate isomerase	41
2.1.1.72	site-specific DNA-methyltransferase (adenine-specific)	40
2.1.2.1	glycine hydroxymethyltransferase	40
2.7.4.22	UMP kinase	40
1.8.1.4	dihydrolipoyl dehydrogenase	39
2.7.2.11	glutamate 5-kinase	39
6.4.1.1	pyruvate carboxylase	39
2.5.1.19	3-phosphoshikimate 1-carboxyvinyltransferase	39
4.2.1.11	phosphopyruvate hydratase	38
2.7.1.69	protein-Npi-phosphohistidine-sugar phosphotransferase	38
2.3.1.191	UDP-3-O-(3-hydroxymyristoyl)glucosamine N-acyltransferase	38
4.2.1.9	dihydroxy-acid dehydratase	37
1.1.1.25	shikimate dehydrogenase	37
1.1.5.3	glycerol-3-phosphate dehydrogenase	37
1.1.1.133	dTDP-4-dehydrorhamnose reductase	37
2.4.1.182	lipid-A-disaccharide synthase	37
5.3.1.1	Triose-phosphate isomerase	36
2.3.1.46	homoserine O-succinyltransferase	36
5.3.1.8	Mannose-6-phosphate isomerase	36
4.2.1.47	GDP-mannose 4,6-dehydratase	36
2.7.7.3	pantetheine-phosphate adenylyltransferase	36
2.7.2.8	acetylglutamate kinase	36
3.2.1.25	beta-mannosidase	36
3.5.4.12	dCMP deaminase	35
1.11.1.9	glutathione peroxidase	35
1.2.1.12	glyceraldehyde-3-phosphate dehydrogenase (phosphorylating)	35

6.3.1.1	Aspartate-ammonia ligase	35
2.7.7.60	2-C-methyl-D-erythritol 4-phosphate cytidylyltransferase	35
1.17.1.2	4-hydroxy-3-methylbut-2-enyl diphosphate reductase	35
3.1.3.11	fructose-bisphosphatase	35
3.2.1.4	cellulase	35
2.1.3.2	aspartate carbamoyltransferase	35
4.2.1.10	3-dehydroquinate dehydratase	34
2.3.3.13	2-isopropylmalate synthase	34
2.7.1.30	glycerol kinase	34
3.6.1.27	undecaprenyl-diphosphate phosphatase	34
2.7.1.148	4-(cytidine 5'-diphospho)-2-C-methyl-D-erythritol kinase	34
2.7.4.14	UMP/CMP kinase	33
2.5.1.90	all-trans-octaprenyl-diphosphate synthase	33
1.17.4.1	ribonucleoside-diphosphate reductase	33
3.1.4.16	2',3'-cyclic-nucleotide 2'-phosphodiesterase	33
2.7.8.8	CDP-diacylglycerol-serine O-phosphatidyltransferase	33
2.5.1.55	3-deoxy-8-phosphooctulonate synthase	33
1.1.1.85	3-isopropylmalate dehydrogenase	32
1.5.1.2	pyrroline-5-carboxylate reductase	32
2.5.1.56	N-acetylneuraminate synthase	32
3.3.1.1	adenosylhomocysteinase	32
2.3.1.8	phosphate acetyltransferase	32
2.7.1.23	NAD+ kinase	32
2.7.8.5	CDP-diacylglycerol-glycerol-3-phosphate 3-phosphatidyltransferase	32
3.1.26.11	tRNase Z	32
1.2.1.38	N-acetyl-gamma-glutamyl-phosphate reductase	32
2.4.2.11	nicotinate phosphoribosyltransferase --> transferred. Now EC 6.3.4.21	32
6.3.1.5	NAD+ synthase	32
3.5.1.108	UDP-3-O-acyl-N-acetylglucosamine deacetylase	32
5.4.99.18	5-(carboxyamino)imidazole ribonucleotide mutase	31
6.3.3.1	phosphoribosylformylglycinamidine cyclo-ligase	31
2.4.2.2	pyrimidine-nucleoside phosphorylase	31
3.2.1.1	alpha-amylase	31
2.7.7.41	phosphatidate cytidylyltransferase	31
3.5.4.2	adenine deaminase	31
4.2.1.7	altronate dehydratase	31
2.6.1.42	branched-chain-amino-acid transaminase	31
1.5.1.20	methylenetetrahydrofolate reductase [NAD(P)H]	31
2.4.2.8	hypoxanthine phosphoribosyltransferase	31
5.3.1.12	Glucuronate isomerase	30
4.1.99.12	3,4-dihydroxy-2-butanone-4-phosphate synthase	30
1.1.1.262	4-hydroxythreonine-4-phosphate dehydrogenase	30
3.5.4.3	guanine deaminase	30
6.1.1.22	Asparagine-tRNA ligase	30
2.4.2.10	orotate phosphoribosyltransferase	30
2.7.1.40	pyruvate kinase	30
3.2.1.31	beta-glucuronidase	29
4.1.1.23	Orotidine-5'-phosphate decarboxylase	29
3.5.1.1	asparaginase	29
5.4.99.5	Chorismate mutase	29

3.6.1.19	nucleoside-triphosphate diphosphatase	29
1.4.3.16	L-aspartate oxidase	29
3.5.4.5	cytidine deaminase	29
3.6.3.34	iron-chelate-transporting ATPase	29
3.2.2.9	adenosylhomocysteine nucleosidase	29
2.4.1.83	dolichyl-phosphate beta-D-mannosyltransferase	28
2.7.1.45	2-dehydro-3-deoxygluconokinase	28
2.7.1.21	thymidine kinase	28
2.5.1.15	dihydropteroate synthase	28
4.2.2.2	pectate lyase	28
4.6.1.12	2-C-methyl-D-erythritol 2,4-cyclodiphosphate synthase	28
5.4.99.2	Methylmalonyl-CoA mutase	28
3.1.21.2	deoxyribonuclease IV (phage-T4-induced)	28
3.6.3.17	monosaccharide-transporting ATPase	28
3.2.2.27	uracil-DNA glycosylase	28
2.3.1.182	(R)-citramalate synthase	28
2.7.2.7	butyrate kinase	27
2.4.2.7	adenine phosphoribosyltransferase	27
2.6.1.52	phosphoserine transaminase	27
2.5.1.72	quinolinate synthase	27
3.1.3.1	alkaline phosphatase	27
4.1.1.65	phosphatidylserine decarboxylase	27
1.1.1.38	malate dehydrogenase (oxaloacetate-decarboxylating)	27
4.1.2.5	L-threonine aldolase	27
3.1.22.4	crossover junction endodeoxyribonuclease	26
1.1.1.57	fructuronate reductase	26
6.3.4.15	Biotin-[acetyl-CoA-carboxylase] ligase	26
3.1.3.18	phosphoglycolate phosphatase	26
6.3.2.6	phosphoribosylaminoimidazolesuccinocarboxamide synthase	26
2.7.1.90	diphosphate-fructose-6-phosphate 1-phosphotransferase	25
3.1.11.2	exodeoxyribonuclease III	25
2.7.1.5	rhamnulokinase	25
3.6.3.31	polyamine-transporting ATPase	24
1.4.1.16	diaminopimelate dehydrogenase	24
2.7.1.31	glycerate 3-kinase	24
6.3.3.2	5-Formyltetrahydrofolate cyclo-ligase	24
1.1.1.3	homoserine dehydrogenase	24
2.1.2.10	aminomethyltransferase	24
4.2.1.75	uroporphyrinogen-III synthase	24
2.4.2.3	uridine phosphorylase	24
1.1.1.271	GDP-L-fucose synthase	23
4.3.1.3	histidine ammonia-lyase	23
6.3.4.5	Argininosuccinate synthase	23
2.8.1.6	biotin synthase	23
4.3.1.17	L-Serine ammonia-lyase	23
2.5.1.54	3-deoxy-7-phosphoheptulonate synthase	23
2.7.1.130	tetraacyldisaccharide 4'-kinase	23
2.1.2.11	3-methyl-2-oxobutanoate hydroxymethyltransferase	23
5.3.1.16	1-(5-phosphoribosyl)-5-[(5-phosphoribosylamino)methylideneamino]imidazole-4-carboxamide isomerase	23

3.4.11.2	membrane alanyl aminopeptidase	23
4.1.2.9	phosphoketolase	23
4.6.1.1	adenylate cyclase	22
2.4.2.22	xanthine phosphoribosyltransferase	22
2.7.1.33	pantothenate kinase	22
3.4.23.36	Signal peptidase II	22
2.7.7.9	UTP-glucose-1-phosphate uridylyltransferase	22
2.7.4.1	polyphosphate kinase	22
2.5.1.78	6,7-dimethyl-8-ribityllumazine synthase	22
2.4.1.20	cellobiose phosphorylase	22
4.1.1.49	phosphoenolpyruvate carboxykinase (ATP)	22
5.4.2.2	phosphoglucomutase (alpha-D-glucose-1,6-bisphosphate-dependent)	22
6.4.1.3	propionyl-CoA carboxylase	21
2.7.7.38	3-deoxy-manno-octulosonate cytidylyltransferase	21
1.1.1.58	tagaturonate reductase	21
1.1.1.290	4-phosphoerythronate dehydrogenase	21
3.5.1.16	acetylornithine deacetylase	21
2.3.1.51	1-acylglycerol-3-phosphate O-acyltransferase	21
5.3.1.14	L-Rhamnose isomerase	21
5.1.3.4	L-ribulose-5-phosphate 4-epimerase	20
6.3.2.1	Pantoate-beta-alanine ligase	20
2.7.9.2	pyruvate, water dikinase	20
2.7.1.71	shikimate kinase	20
2.7.1.24	dephospho-CoA kinase	20
3.2.1.24	alpha-mannosidase	20
5.3.1.25	L-Fucose isomerase	19
4.2.1.19	imidazoleglycerol-phosphate dehydratase	19
4.1.1.48	indole-3-glycerol-phosphate synthase	19
2.6.99.2	pyridoxine 5'-phosphate synthase	19
5.4.2.7	phosphopentomutase	18
4.1.2.19	rhamnulose-1-phosphate aldolase	18
1.1.1.42	isocitrate dehydrogenase (NADP+)	18
1.1.1.86	ketol-acid reductoisomerase	18
3.5.1.25	N-acetylglucosamine-6-phosphate deacetylase	18
1.8.98.1	CoB-CoM heterodisulfide reductase	18
2.5.1.3	thiamine-phosphate diphosphorylase	17
3.2.1.10	oligo-1,6-glucosidase	17
4.2.1.126	N-acetylmuramic acid 6-phosphate etherase	17
4.1.1.82	phosphonopyruvate decarboxylase	17
3.1.3.25	inositol-phosphate phosphatase	17
1.1.1.169	2-dehydropantoate 2-reductase	17
5.4.4.2	Isochorismate synthase	17
1.4.1.1	alanine dehydrogenase	17
2.7.6.3	2-amino-4-hydroxy-6-hydroxymethyldihydropteridine diphosphokinase	17
1.5.1.7	saccharopine dehydrogenase (NAD+, L-lysine-forming)	17
2.4.2.19	nicotinate-nucleotide diphosphorylase (carboxylating)	17
1.2.1.3	aldehyde dehydrogenase (NAD+)	17
5.4.2.10	phosphoglucosamine mutase	17
3.1.3.2	acid phosphatase	17
2.7.1.35	pyridoxal kinase	16

2.7.4.6	nucleoside-diphosphate kinase	16
2.3.3.1	citrate (Si)-synthase	16
4.2.1.8	Mannonate dehydratase	16
3.5.1.18	succinyl-diaminopimelate desuccinylase	16
1.2.1.2	formate dehydrogenase	16
2.1.3.3	ornithine carbamoyltransferase	16
3.6.1.13	ADP-ribose diphosphatase	16
1.1.1.37	malate dehydrogenase	16
2.4.2.18	anthranilate phosphoribosyltransferase	16
4.2.1.45	CDP-glucose 4,6-dehydratase	15
2.7.7.19	polynucleotide adenylyltransferase	15
2.3.1.9	acetyl-CoA C-acetyltransferase	15
2.1.1.148	thymidylate synthase (FAD)	15
1.1.3.15	(S)-2-hydroxy-acid oxidase	15
6.4.1.2	acetyl-CoA carboxylase	15
3.5.3.12	agmatine deiminase	15
2.3.1.19	phosphate butyryltransferase	15
4.2.3.12	6-pyruvoyltetrahydropterin synthase	15
1.1.1.29	glycerate dehydrogenase	15
1.18.6.1	nitrogenase	15
2.7.8.30	undecaprenyl-phosphate 4-deoxy-4-formamido-L-arabinose transferase --> deleted. Now EC 2.4.2.53	14
2.3.1.29	glycine C-acetyltransferase	14
3.5.1.53	N-carbamoylputrescine amidase	14
3.6.3.30	Fe3+-transporting ATPase	14
2.5.1.9	riboflavin synthase	14
3.2.1.91	cellulose 1,4-beta-cellobiosidase (non-reducing end)	14
1.5.1.3	dihydrofolate reductase	14
2.7.2.2	carbamate kinase	14
2.7.7.4	sulfate adenylyltransferase	14
3.1.3.10	glucose-1-phosphatase	14
2.1.1.14	5-methyltetrahydropteroyltriglutamate-homocysteine S-methyltransferase	14
3.1.1.11	pectinesterase	14
1.2.7.1	pyruvate synthase	14
5.3.1.17	5-dehydro-4-deoxy-D-glucuronate isomerase	14
3.5.3.11	agmatinase	13
4.1.1.81	threonine-phosphate decarboxylase	13
2.7.7.39	glycerol-3-phosphate cytidylyltransferase	13
2.7.4.9	dTMP kinase	13
2.1.1.195	cobalt-precorrin-5B (C1)-methyltransferase	13
5.3.1.5	Xylose isomerase	13
6.3.5.10	adenosylcobyric acid synthase (glutamine-hydrolysing)	13
2.7.7.18	nicotinate-nucleotide adenylyltransferase	13
3.1.3.45	3-deoxy-manno-octulosonate-8-phosphatase	13
2.7.7.63	lipoate-protein ligase	12
4.1.1.32	phosphoenolpyruvate carboxykinase (GTP)	12
5.3.1.23	S-methyl-5-thioribose-1-phosphate isomerase	12
1.1.1.28	D-lactate dehydrogenase	12
3.6.1.22	NAD+ diphosphatase	12
5.3.3.2	isopentenyl-diphosphate DELTA-isomerase	12
3.6.1.54	UDP-2,3-diacylglucosamine diphosphatase	12

1.15.1.1	superoxide dismutase	12
4.1.1.11	aspartate 1-decarboxylase	12
2.7.7.43	N-acylneuraminate cytidylyltransferase	12
2.3.3.14	homocitrate synthase	12
1.1.1.336	UDP-N-acetyl-D-mannosamine dehydrogenase	11
1.2.99.2	carbon-monoxide dehydrogenase (acceptor)	11
5.3.1.28	D-sedoheptulose 7-phosphate isomerase	11
5.1.3.20	ADP-glyceromanno-heptose 6-epimerase	11
4.2.1.6	galactonate dehydratase	11
4.2.1.113	o-succinylbenzoate synthase	11
3.2.1.26	beta-fructofuranosidase	11
3.5.4.4	adenosine deaminase	10
2.7.7.12	UDP-glucose-hexose-1-phosphate uridylyltransferase	10
5.3.1.24	phosphoribosylanthranilate isomerase	10
1.3.1.12	prephenate dehydrogenase	10
2.7.7.33	glucose-1-phosphate cytidylyltransferase	10
2.4.2.21	nicotinate-nucleotide-dimethylbenzimidazole phosphoribosyltransferase	10
1.1.1.77	lactaldehyde reductase	10
4.1.1.19	Arginine decarboxylase	10
1.6.1.2	NAD(P)+ transhydrogenase (Re/Si-specific)	10
5.4.3.8	glutamate-1-semialdehyde 2,1-aminomutase	10
2.1.3.11	N-succinylornithine carbamoyltransferase	10
2.5.1.17	cob(I)yrinic acid a,c-diamide adenosyltransferase	10
2.4.2.28	S-methyl-5'-thioadenosine phosphorylase	10
1.1.1.44	phosphogluconate dehydrogenase (NADP+-dependent, decarboxylating)	10
4.3.1.1	aspartate ammonia-lyase	10
2.7.7.23	UDP-N-acetylglucosamine diphosphorylase	10
3.2.1.45	glucosylceramidase	10
2.7.8.26	adenosylcobinamide-GDP ribazoletransferase	10
2.3.1.31	homoserine O-acetyltransferase	10
3.1.26.12	ribonuclease E	10
1.1.1.41	isocitrate dehydrogenase (NAD+)	9
2.2.1.9	2-succinyl-5-enolpyruvyl-6-hydroxy-3-cyclohexene-1-carboxylic-acid synthase	9
5.1.3.8	N-Acylglucosamine 2-epimerase	9
4.1.1.18	Lysine decarboxylase	9
2.6.1.11	acetylornithine transaminase	9
4.4.1.21	S-ribosylhomocysteine lyase	9
1.5.1.12	1-pyrroline-5-carboxylate dehydrogenase --> transferred to EC 1.2.1.88	9
5.5.1.4	inositol-3-phosphate synthase	9
1.1.1.157	3-hydroxybutyryl-CoA dehydrogenase	9
1.1.1.122	D-threo-aldose 1-dehydrogenase	9
3.5.1.10	formyltetrahydrofolate deformylase	9
4.2.1.51	prephenate dehydratase	9
2.7.11.1	non-specific serine/threonine protein kinase	8
4.2.3.3	methylglyoxal synthase	8
3.5.2.7	imidazolonepropionase	8
2.6.1.37	2-aminoethylphosphonate-pyruvate transaminase	8
4.1.99.1	tryptophanase	8
1.3.99.5	3-oxo-5alpha-steroid 4-dehydrogenase (acceptor)	8
3.5.2.2	dihydropyrimidinase	8

5.1.1.13	Aspartate racemase	8
4.4.1.5	lactoylglutathione lyase	8
1.2.4.4	3-methyl-2-oxobutanoate dehydrogenase (2-methylpropanoyl-transferring)	8
3.2.1.37	xylan 1,4-beta-xylosidase	8
3.4.11.1	leucyl aminopeptidase	8
1.7.2.2	nitrite reductase (cytochrome; ammonia-forming)	8
1.3.99.23	all-trans-retinol 13,14-reductase	8
1.5.3.1	sarcosine oxidase	7
6.6.1.2	cobaltochelatase	7
4.1.3.39	4-hydroxy-2-oxovalerate aldolase	7
4.1.1.33	Diphosphomevalonate decarboxylase	7
4.2.1.1	carbonate dehydratase	7
2.8.4.1	coenzyme-B sulfoethylthiotransferase	7
2.3.1.12	dihydrolipoyllysine-residue acetyltransferase	7
1.1.1.81	hydroxypyruvate reductase	7
1.8.99.2	adenylyl-sulfate reductase	7
3.5.3.6	arginine deiminase	7
5.4.3.3	beta-lysine 5,6-aminomutase	7
6.2.1.5	Succinate-CoA ligase (ADP-forming)	6
1.1.1.125	2-deoxy-D-gluconate 3-dehydrogenase	6
1.2.4.1	pyruvate dehydrogenase (acetyl-transferring)	6
3.1.3.71	2-phosphosulfolactate phosphatase	6
2.7.1.15	ribokinase	6
6.3.1.10	adenosylcobinamide-phosphate synthase	6
2.4.1.157	1,2-diacylglycerol 3-glucosyltransferase	6
3.1.1.61	protein-glutamate methylesterase	6
4.2.1.24	porphobilinogen synthase	6
2.1.1.132	precorrin-6B C5,15-methyltransferase (decarboxylating)	6
3.2.1.18	exo-alpha-sialidase	6
2.5.1.16	spermidine synthase	6
1.1.1.140	sorbitol-6-phosphate 2-dehydrogenase	6
1.1.1.40	malate dehydrogenase (oxaloacetate-decarboxylating) (NADP+)	6
6.6.1.1	magnesium chelatase	6
6.2.1.26	O-succinylbenzoate-CoA ligase	6
2.6.1.13	ornithine aminotransferase	6
2.3.1.117	2,3,4,5-tetrahydropyridine-2,6-dicarboxylate N-succinyltransferase	6
4.3.1.12	ornithine cyclodeaminase	6
2.2.1.2	transaldolase	6
1.3.8.1	short-chain acyl-CoA dehydrogenase	6
4.1.2.17	L-fuculose-phosphate aldolase	5
5.4.2.6	beta-Phosphoglucomutase	5
3.2.2.4	AMP nucleosidase	5
1.2.99.5	formylmethanofuran dehydrogenase	5
2.7.1.16	ribulokinase	5
2.1.1.10	homocysteine S-methyltransferase	5
1.1.1.14	L-iditol 2-dehydrogenase	5
4.99.1.3	sirohydrochlorin cobaltochelatase	5
4.2.1.49	Urocanate hydratase	5
3.1.1.1	carboxylesterase	5
4.1.2.25	dihydroneopterin aldolase	5

1.6.99.5	NADH dehydrogenase (quinone)	5
5.4.3.2	lysine 2,3-aminomutase	5
3.6.1.31	phosphoribosyl-ATP diphosphatase	5
3.2.1.50	alpha-N-acetylglucosaminidase	5
5.3.1.4	L-Arabinose isomerase	5
2.3.1.1	amino-acid N-acetyltransferase	5
2.7.7.73	sulfur carrier protein ThiS adenylyltransferase	5
2.7.1.39	homoserine kinase	5
2.6.1.62	adenosylmethionine-8-amino-7-oxononanoate transaminase	5
1.1.1.60	2-hydroxy-3-oxopropionate reductase	5
5.1.3.9	N-Acylglucosamine-6-phosphate 2-epimerase	5
1.3.3.4	protoporphyrinogen oxidase	5
4.1.1.17	ornithine decarboxylase	4
1.3.1.31	2-enoate reductase	4
1.1.1.26	glyoxylate reductase	4
3.1.11.1	exodeoxyribonuclease I	4
3.2.1.39	glucan endo-1,3-beta-D-glucosidase	4
3.2.1.54	cyclomaltodextrinase	4
5.4.1.2	Precorrin-8X methylmutase	4
1.17.1.4	xanthine dehydrogenase	4
2.7.1.36	mevalonate kinase	4
4.1.2.22	fructose-6-phosphate phosphoketolase	4
4.3.1.19	threonine ammonia-lyase	4
3.4.11.5	prolyl aminopeptidase	4
2.7.1.50	hydroxyethylthiazole kinase	4
4.2.1.32	L(+)-Tartrate dehydratase	4
4.1.1.37	Uroporphyrinogen decarboxylase	4
2.3.3.5	2-methylcitrate synthase	4
3.1.3.3	phosphoserine phosphatase	4
2.7.6.2	thiamine diphosphokinase	4
4.2.1.44	Myo-inosose-2 dehydratase	4
2.1.1.133	Precorrin-4 C11-methyltransferase	4
6.1.1.23	aspartate-tRNAAsn ligase	4
2.3.1.61	dihydrolipoyllysine-residue succinyltransferase	4
3.1.1.5	lysophospholipase	4
1.1.1.272	D-2-hydroxyacid dehydrogenase (NADP+)	3
6.2.1.12	4-Coumarate-CoA ligase	3
1.16.3.1	ferroxidase	3
2.1.1.245	5-methyltetrahydrosarcinapterin:corrinoid/iron-sulfur protein Co-methyltransferase	3
4.1.3.36	1,4-dihydroxy-2-naphthoyl-CoA synthase	3
3.6.1.7	acylphosphatase	3
3.2.2.21	DNA-3-methyladenine glycosylase II	3
3.5.3.1	arginase	3
3.5.4.13	dCTP deaminase	3
6.3.5.7	glutaminyl-tRNA synthase (glutamine-hydrolysing)	3
3.1.1.3	triacylglycerol lipase	3
3.4.21.107	peptidase Do	3
2.1.1.86	tetrahydromethanopterin S-methyltransferase	3
2.6.1.76	diaminobutyrate-2-oxoglutarate transaminase	3
1.4.1.2	glutamate dehydrogenase	3

1.2.1.59	glyceraldehyde-3-phosphate dehydrogenase (NAD(P)+) (phosphorylating)	3
4.1.3.3	N-acetylneuraminate lyase	3
2.7.1.107	diacylglycerol kinase (ATP dependent)	3
1.1.1.1	alcohol dehydrogenase	3
3.1.3.15	histidinol-phosphatase	3
2.1.1.80	protein-glutamate O-methyltransferase	3
2.7.1.56	1-phosphofructokinase	3
3.2.1.35	hyaluronoglucosaminidase	3
3.5.4.1	cytosine deaminase	3
1.1.1.36	acetoacetyl-CoA reductase	3
2.7.9.3	selenide, water dikinase	3
2.1.1.131	precorrin-3B C17-methyltransferase	3
1.8.1.7	glutathione-disulfide reductase	3
1.2.7.7	3-methyl-2-oxobutanoate dehydrogenase (ferredoxin)	3
4.4.1.11	methionine gamma-lyase	3
4.2.1.55	3-Hydroxybutyryl-CoA dehydratase	3
4.1.1.12	aspartate 4-decarboxylase	3
3.1.6.1	arylsulfatase	3
1.3.1.44	trans-2-enoyl-CoA reductase (NAD+)	3
3.1.1.47	1-alkyl-2-acetylglycerophosphocholine esterase	3
4.1.1.44	4-carboxymuconolactone decarboxylase	3
3.5.1.44	protein-glutamine glutaminase	3
3.6.3.28	phosphonate-transporting ATPase	2
2.7.1.144	tagatose-6-phosphate kinase	2
2.5.1.18	glutathione transferase	2
1.12.98.1	coenzyme F420 hydrogenase	2
4.3.1.2	methylaspartate ammonia-lyase	2
3.5.2.10	creatininase	2
2.8.3.5	3-oxoacid CoA-transferase	2
2.7.1.51	L-fuculokinase	2
2.7.7.65	diguanylate cyclase	2
1.3.1.2	dihydropyrimidine dehydrogenase (NADP+)	2
6.4.1.4	methylcrotonoyl-CoA carboxylase	2
3.4.25.1	proteasome endopeptidase complex	2
1.1.1.34	hydroxymethylglutaryl-CoA reductase (NADPH)	2
5.4.99.1	Methylaspartate mutase	2
2.7.7.15	choline-phosphate cytidylyltransferase	2
3.8.1.2	(S)-2-haloacid dehalogenase	2
1.2.7.5	aldehyde ferredoxin oxidoreductase	2
4.2.1.40	glucarate dehydratase	2
2.5.1.61	hydroxymethylbilane synthase	2
3.5.1.5	urease	2
3.5.2.6	beta-lactamase	2
5.1.3.15	glucose-6-phosphate 1-epimerase	2
1.1.1.103	L-threonine 3-dehydrogenase	2
1.7.1.7	GMP reductase	2
4.1.1.41	Methylmalonyl-CoA decarboxylase	2
4.4.1.15	D-cysteine desulfhydrase	2
2.7.8.25	triphosphoribosyl-dephospho-CoA synthase --> deleted. Now EC 2.4.2.52	2
2.6.1.82	putrescine aminotransferase	2

4.1.1.15	Glutamate decarboxylase	2
1.8.4.8	phosphoadenylyl-sulfate reductase (thioredoxin)	2
4.2.2.3	poly(beta-D-mannuronate) lyase	2
2.4.1.12	cellulose synthase (UDP-forming)	2
4.1.2.40	tagatose-bisphosphate aldolase	2
3.6.3.25	sulfate-transporting ATPase	2
1.4.7.1	glutamate synthase (ferredoxin)	2
2.7.3.9	phosphoenolpyruvate-protein phosphotransferase	2
2.7.8.17	UDP-N-acetylglucosamine-lysosomal-enzyme N-acetylglucosaminephosphotransferase	2
2.5.1.30	heptaprenyl diphosphate synthase	1
2.3.1.101	formylmethanofuran-tetrahydromethanopterin N-formyltransferase	1
3.8.1.3	haloacetate dehalogenase	1
6.3.2.19	Ubiquitin-protein ligase	1
4.1.1.74	Indolepyruvate decarboxylase	1
1.9.3.1	cytochrome-c oxidase	1
5.1.3.10	CDP-paratose 2-epimerase	1
3.2.1.86	6-phospho-beta-glucosidase	1
4.1.1.50	adenosylmethionine decarboxylase	1
1.1.3.9	galactose oxidase	1
3.1.4.3	phospholipase C	1
3.6.3.40	teichoic-acid-transporting ATPase	1
1.3.99.12	2-methylacyl-CoA dehydrogenase	1
4.1.1.61	4-Hydroxybenzoate decarboxylase	1
3.1.4.17	3',5'-cyclic-nucleotide phosphodiesterase	1
1.12.99.6	hydrogenase (acceptor)	1
1.2.1.31	L-aminoadipate-semialdehyde dehydrogenase	1
2.4.1.7	sucrose phosphorylase	1
3.5.1.2	glutaminase	1
2.5.1.68	(2Z,6E)-farnesyl diphosphate synthase	1
2.3.1.170	6'-deoxychalcone synthase	1
1.1.1.17	mannitol-1-phosphate 5-dehydrogenase	1
1.2.3.3	pyruvate oxidase	1
3.5.5.1	nitrilase	1
3.1.3.6	3'-nucleotidase	1
2.4.2.4	thymidine phosphorylase	1
2.5.1.26	alkylglycerone-phosphate synthase	1
1.1.1.130	3-dehydro-L-gulonate 2-dehydrogenase	1
2.5.1.48	cystathionine gamma-synthase	1
6.1.1.13	D-Alanine-poly(phosphoribitol) ligase	1
1.4.1.21	aspartate dehydrogenase	1
3.1.3.7	3'(2'),5'-bisphosphate nucleotidase	1
2.4.1.141	N-acetylglucosaminyldiphosphodolichol N-acetylglucosaminyltransferase	1
5.2.1.8	Peptidylprolyl isomerase	1
1.7.99.4	nitrate reductase	1
1.18.1.1	rubredoxin-NAD+ reductase	1
1.12.7.2	ferredoxin hydrogenase	1
3.1.1.41	cephalosporin-C deacetylase	1
5.1.99.4	alpha-Methylacyl-CoA racemase	1
3.2.1.49	alpha-N-acetylgalactosaminidase	1
3.4.24.11	neprilysin	1

1.1.1.88	hydroxymethylglutaryl-CoA reductase	1
1.13.12.16	nitronate monooxygenase	1
2.7.12.2	mitogen-activated protein kinase kinase	1
1.2.1.70	glutamyl-tRNA reductase	1
2.8.3.17	cinnamoyl-CoA:phenyllactate CoA-transferase	1
4.1.1.35	UDP-glucuronate decarboxylase	1
3.2.2.8	ribosylpyrimidine nucleosidase	1
2.6.1.19	4-aminobutyrate-2-oxoglutarate transaminase	1
2.3.3.10	hydroxymethylglutaryl-CoA synthase	1
3.1.13.4	poly(A)-specific ribonuclease	1
1.1.1.215	gluconate 2-dehydrogenase	1
1.1.1.67	mannitol 2-dehydrogenase	1
3.1.1.17	gluconolactonase	1
3.5.1.54	allophanate hydrolase	**1**
1.2.1.10	acetaldehyde dehydrogenase (acetylating)	1
6.1.1.24	glutamate-tRNAGln ligase	1
3.2.1.84	glucan 1,3-alpha-glucosidase	1
2.4.1.11	glycogen(starch) synthase	1
2.7.1.52	fucokinase	1
2.7.8.7	holo-[acyl-carrier-protein] synthase	1
4.4.1.1	cystathionine gamma-lyase	1
4.1.1.39	Ribulose-bisphosphate carboxylase	1
3.4.24.16	neurolysin	1
1.1.1.251	galactitol-1-phosphate 5-dehydrogenase	1
2.1.2.3	phosphoribosylaminoimidazolecarboxamide formyltransferase	1
1.1.1.30	3-hydroxybutyrate dehydrogenase	1
4.1.1.28	aromatic-L-amino-acid decarboxylase	1
2.7.1.12	gluconokinase	1
3.6.3.8	Ca2+-transporting ATPase	1
6.5.1.1	DNA ligase (ATP)	1
1.1.1.53	3alpha(or 20beta)-hydroxysteroid dehydrogenase	1
3.1.1.31	6-phosphogluconolactonase	1
3.2.1.46	galactosylceramidase	1
4.3.1.7	ethanolamine ammonia-lyase	1
3.5.1.4	amidase	1
3.6.1.11	exopolyphosphatase	1
3.6.3.27	phosphate-transporting ATPase	1

Supplementary Table S4. List showing abundance and diversity of all six classes of CAZyme families in HF cross rumen metagenome

CAZy family AA[1]	No of contigs	CAZy family CBM[2]	No of contigs	CAZy family CE[3]	No of contigs	CAZy family GH[4]	No of contigs	CAZy family GT[5]	No of contigs	CAZy family PL[6]	No of contigs
AA6	50	CBM37	1241	CE10	361	GH3	993	GT2	2795	PL1	225
AA5	50	CBM50	962	CE1	390	GH2	927	GT4	1207	PL11	117
AA4	6	CBM6	482	CE11	291	GH43	739	GT51	403	PL9	31
AA10	5	CBM48	383	CE4	198	GH53	591	GT28	238	PL10	29
AA7	3	CBM32	328	CE9	199	GH13	498	GT41	197	PL12	23
AA3	1	CBM2	254	CE6	164	GH23	456	GT8	193	PL6	15
		CBM56	181	CE8	111	GH39	422	GT35	169	PL8	10
		CBM13	178	CE12	71	GH5	403	GT26	160	PL4	8
		CBM12	147	CE7	62	GH92	330	GT9	150	PL17	6
		CBM35	138	CE15	55	GH31	285	GT30	139	PL21	6
		CBM9	86	CE2	38	GH95	272	GT5	139	PL15	4
		CBM20	45	CE14	31	GH33	209	GT82	81	PL14	2
		CBM4	82	CE3	4	GH97	208	GT19	74	PL22	2
		CBM57	75			GH51	201	GT47	66	PL3	1
		CBM5	59			GH4	199	GT32	52	PL7	1
		CBM51	54			GH78	196	GT3	48		
		CBM27	44			GH9	183	GT66	44		
		CBM3	33			GH18	158	GT11	31		
		CBM25	26			GH20	151	GT49	31		
		CBM22	24			GH105	149	GT83	30		
		CBM58	20			GH28	149	GT1	26		
		CBM16	19			GH77	149	GT55	19		
		CBM26	19			GH16	147	GT87	17		
		CBM66	16			GH36	148	GT10	13		
		CBM47	15			GH25	146	GT14	11		
		CBM34	7			GH29	142	GT25	7		
		CBM38	7			GH73	135	GT34	5		
		CBM18	5			GH32	133	GT39	5		
		CBM67	5			GH94	132	GT81	3		
		CBM42	3			GH109	129	GT76	2		
		CBM14	2			GH1	126	GT90	2		
		CBM23	2			GH10	118	GT20	1		
		CBM10	1			GH115	105	GT29	1		
		CBM45	1			GH106	102	GT48	1		
		CBM60	1			GH72	98	GT56	1		
						GH127	97	GT71	1		
						GH50	73	GT80	1		
						GH30	71	GT22	1		

						GH57	71	GT50	1		
						GH26	67	GT77	1		
						GH8	66				
						GH35	65				
						GH128	61				
						GH67	57				
						GH130	56				
						GH65	54				
						GH27	50				
						GH38	46				
						GH99	46				
						GH17	39				
						GH24	35				
						GH12	31				
						GH98	30				
						GH54	28				
						GH89	28				
						GH66	27				
						GH120	26				
						GH42	26				
						GH74	26				
						GH84	21				
						GH125	19				
						GH76	19				
						GH129	18				
						GH19	18				
						GH11	17				
						GH123	17				
						GH88	17				
						GH63	16				
						GH87	16				
						GH112	11				
						GH45	11				
						GH48	11				
						GH113	10				
						GH93	9				
						GH116	8				
						GH103	7				
						GH6	6				
						GH102	5				
						GH117	5				
						GH121	5				
						GH44	5				

						GH91	5				
						GH104	4				
						GH59	4				
						GH108	3				
						GH110	3				
						GH55	3				
						GH70	3				
						GH58	2				
						GH101	1				
						GH107	1				
						GH124	1				
						GH132	1				
						GH47	1				
						GH81	1				
						GH85	1				

[1]AA:auxiliary activities
[2]CBM:carbohydrate-binding modules
[3]CE:carbohydrate esterases
[4]GH:glycoside hydrolases
[5]GT:glycosyltransferases
[6]PL:polysaccharide lyases

Supplementary Figure S1. Krona chart illustrating distribution of the functional categories indicating the percentages of reads with predicted protein functions using SEED subsystem database

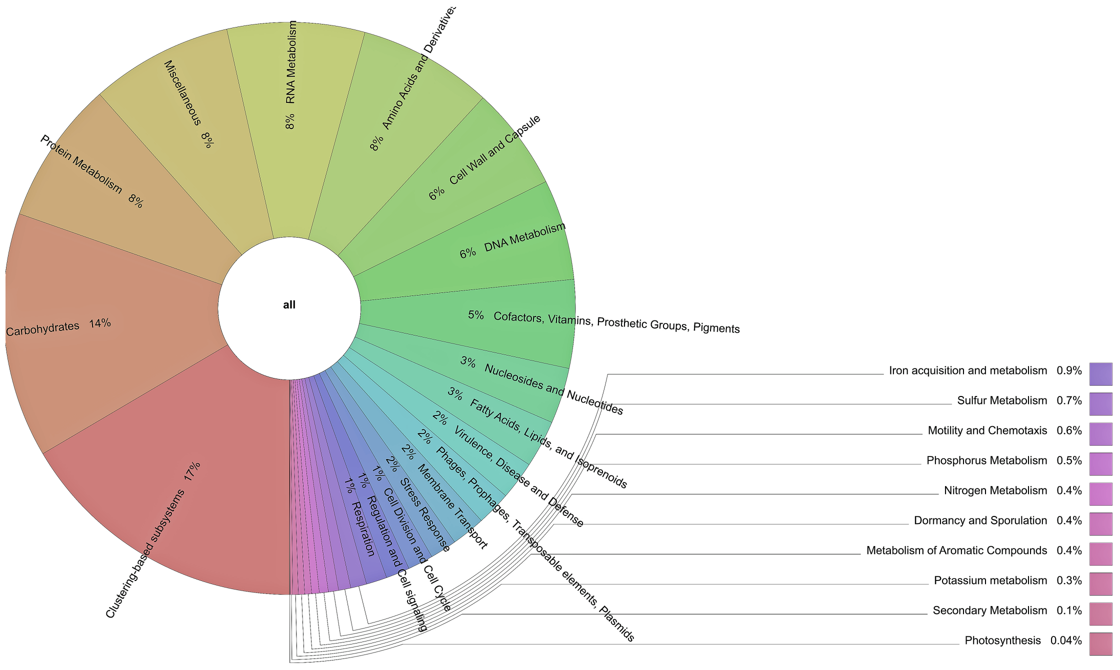

Supplementary Figure S2. KEGG map showing the metabolic pathways identified in HF cross rumen metagenome using KEGG mapper view, where blue lines highlight the pathways present in metadata

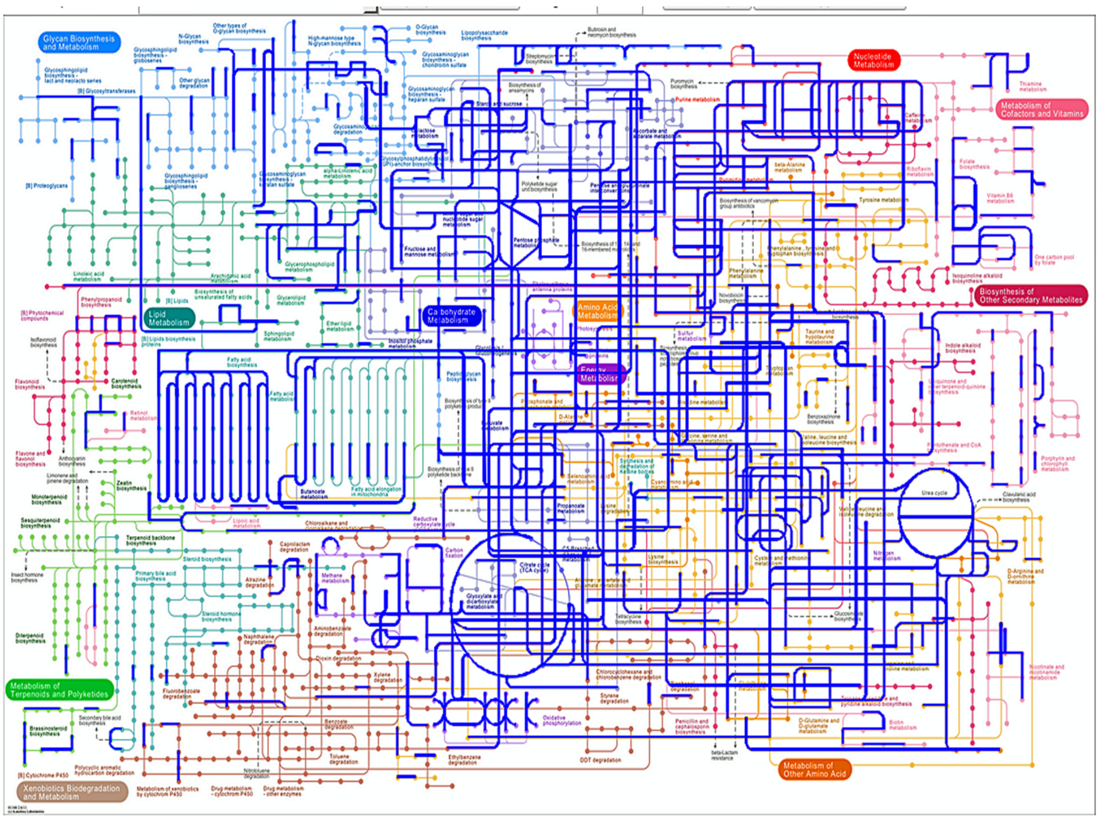

Supplement: Supplementary file 1 — Additional file 1. Additional tables and figures. [file 13568_2016_310_MOESM1_ESM.pdf]
